# Untargeted metabolomics reveals distinct metabolic reprogramming in endothelial cells co-cultured with CSC and non-CSC prostate cancer cell subpopulations

**DOI:** 10.1371/journal.pone.0192175

**Published:** 2018-02-21

**Authors:** Anusha Jayaraman, Praveen Kumar, Silvia Marin, Pedro de Atauri, Francesca Mateo, Timothy M. Thomson, Josep J. Centelles, Stewart F. Graham, Marta Cascante

**Affiliations:** 1 Department of Biochemistry and Molecular Biomedicine, Faculty of Biology, Universitat de Barcelona, Barcelona, Spain; 2 Beaumont Health System, Beaumont Research Institute, Royal Oak, Michigan, United States of America; 3 Centro de Investigacion Biomedica en Red de Enfermedades Hepaticas y Digestivas (CIBEREHD), Instituto de Salud Carlos III (ISCIII), Madrid, Spain; 4 Department of Cell Biology, Molecular Biology Institute of Barcelona, National Research Council (IBMB-CSIC), Barcelona, Spain; Southern Illinois University School of Medicine, UNITED STATES

## Abstract

Tumour angiogenesis is an important hallmark of cancer and the study of its metabolic adaptations, downstream to any cellular change, can reveal attractive targets for inhibiting cancer growth. In the tumour microenvironment, endothelial cells (ECs) interact with heterogeneous tumour cell types that drive angiogenesis and metastasis. In this study we aim to characterize the metabolic alterations in ECs influenced by the presence of tumour cells with extreme metastatic abilities. Human umbilical vein endothelial cells (HUVECs) were subjected to different microenvironmental conditions, such as the presence of highly metastatic PC-3M and highly invasive PC-3S prostate cancer cell lines, in addition to the angiogenic activator vascular endothelial growth factor (VEGF), under normoxia. Untargeted high resolution liquid chromatography-mass spectrometry (LC-MS) based metabolomics revealed significant metabolite differences among the various conditions and a total of 25 significantly altered metabolites were identified including acetyl L-carnitine, NAD+, hypoxanthine, guanine and oleamide, with profile changes unique to each of the experimental conditions. Biochemical pathway analysis revealed the importance of fatty acid oxidation and nucleotide salvage pathways. These results provide a global metabolic preview that could help in selectively targeting the ECs aiding in either cancer cell invasion or metastasis in the heterogeneous tumour microenvironment.

## Introduction

Tumour microenvironment is a perfectly designed niche for cancer cells, in that they have acquired the ability to break all the cellular rules and hijack the stromal cells for their survival and propagation [1]. Tumour vascularization is considered as an essential system for cancer proliferation and is crucial for providing oxygen and nutrients for survival, invasion and enables metastasis to other distal locations [2]. Endothelial cells (ECs), like other stromal cells such as cancer-associated fibroblasts and macrophages, can be reprogrammed by tumour-released factors inducing angiogenesis [2]. As our knowledge of tumour angiogenesis expands, its potential as an alternative target for cancer treatment is being increasingly explored and could be considered complementary to the conventional treatments that target only the cancer cells [3]. Clinical therapies targeting angiogenesis have been mostly aimed at inhibiting cellular signalling and have only been partially successful [3]. Tumour-released factors can significantly affect the ECs downstream angiogenic signalling, i.e. at the level of cellular metabolism suggesting that they may be attractive targets for anti-cancer therapy [4]. General EC metabolism has been described by some of the main central carbon metabolic pathways to include glycolysis, Kreb’s cycle and pentose phosphate pathway (PPP), while metabolic changes in the tumour-driven EC growth have not as yet been extensively characterized [5]. In order to understand the metabolic changes that affect angiogenesis associated with tumours it is important to choose a method that can focus only on the affected ECs, which is different *in vivo* due to the complexity associated with extracting different types of stromal cells from the tumour tissues. The *in vitro* co-culture method employed in this study intends to explore specifically the tumour-endothelial cell association. Previous studies on *in vitro* endothelial—tumour cell interactions have been performed using different co-culture models and the cellular changes were assessed in gene and protein expression analysis and cellular phenotypes [6–9]. However metabolic changes due to this stromal-tumour cellular interaction are yet to be explored.

In this study we aim for the first time to characterize the global metabolic profile of ECs under the influence of cancer cell sub-populations with differing metastatic abilities. To achieve this we apply a high resolution mass spectrometry—based untargeted metabolomics analysis which involves a generic extraction, chromatographic separation and detection of analyte ions, data pre-processing and analysis, followed by identification of interested metabolites without *a priori* information [10]. Metabolite set enrichment analysis (MSEA) was used to explore the metabolites highly enriched and associated with possible metabolic pathways [11] and the results of metabolite changes and pathway enrichment obtained with each condition are discussed in the following section. These results provide an overall preview of the metabolic plasticity of ECs in the heterogeneous tumour microenvironment, which could be exploited in combined therapies targeting not only the tumour cell reprogramming, but also the metabolic changes of ECs induced by the tumour microenvironment.

## Materials and methods

### Cell culture conditions

Human umbilical vein endothelial cells (HUVECs), purchased from Lonza (CC-2519) were maintained in 1% gelatin coated flasks at 37°C in a humidified atmosphere of 5% CO_2_ and 95% air in MCDB131 (Gibco) complete medium supplemented with recommended quantity of endothelial growth medium SingleQuots (EGM) (Lonza), 10% fetal bovine serum (FBS) (Gibco), 2 mM glutamine (Gibco) and 1% streptomycin (100 μg/mL)/penicillin (100 units/mL) (S/P, Gibco). The prostate cancer cell sub-populations, PC-3M and PC-3S were clonally derived from the human cell line PC-3 [12]. These cells were maintained at 37°C in a 5% CO_2_ atmosphere in RPMI 1640 (Sigma-Aldrich or Biowest) complete medium supplemented with 10 mM glucose and 2 mM glutamine, 10% FBS (PAA Laboratories), 1% pyruvate (1 mM) (Biological Industries), 1% S/P and 1% nonessential amino acids (Biological Industries).

### Co-culture experiments: Conditions and procedure

HUVECs were seeded in 6-well plates (Falcon) in MCDB131 complete medium, and after 6h they were deprived of nutrients/hormones with restricted medium (RM) consisting of DMEM with 5.56 mM glucose (Sigma) supplemented with 2% FBS, 2 mM glutamine and 0.1% streptomycin/penicillin, overnight. Simultaneously, PC-3M and PC-3S cells were seeded in cell culture inserts supported in separate 6-well plates (Falcon) in RPMI complete medium. After 24h all the cells were washed with PBS and RM was added to both HUVECs and PC-3M and PC-3S cells, and inserts placed over the wells for the co-culture incubation. For the VEGF condition, HUVECs were maintained in the 6-well plates in RM supplemented with 30 ng/mL of Human vascular endothelial growth factor 165 (VEGF165, Miltenyi Biotec) and control HUVECs were maintained only with the RM. Following a 24h incubation of the control and the experimental conditions under normoxia (21% O_2_, 5% CO_2_) at 37°C in a humidified atmosphere, the HUVECs in the wells (n = 3 biological replicates) were trypsinized and the pellets stored at -80°C until extraction.

### Sample preparation

500 μL of 50% ice-cold MeOH/water was added to the frozen cell pellets and 25 μL of 0.001 mg/mL of tryptophan D3 (internal standard) was added to all the samples prior to extraction. Samples were mixed using a microplate shaker for 10 min, followed by ultrasonication at 4°C for 20 min and mixed again using a microplate shaker for 10 min. Samples were subsequently centrifuged at 13,000 *g* at 4°C for 20 min and the supernatant collected. The supernatant was evaporated to dryness under vacuum at room temperature and reconstituted in 150 μL of ultra-pure water. The sample extract was filtered using Whatmann syringeless filters (0.2 μm) and the filtrate transferred to a maximum recovery vial for analysis. 5 μL was injected onto the LC/MS (Dionex 3000, Thermo Scientific; n = 3 technical replicates). For quality control, a pooled sample was made from all samples together and injected at intervals of every 10 samples throughout the entire experiment to determine the chromatographic reproducibility of retention times and peak intensities, and was used for fragmentations later [13, 14].

### LTQ—Orbitrap Elite LC/MS analysis

Dionex 3000 Ultra High Performance Liquid Chromatography (UHPLC) coupled with an Orbitrap Elite was used for acquiring the data. The chromatographic system was coupled to the mass spectrometer with a heated electrospray ionization source II (HESII). The optimized HESII conditions for both ESI+ and ESI- were: spray voltage of 3.5 kV; sheath gas flow rate (N_2_), 60 units; auxiliary gas flow rate, 45; sweep gas flow rate, 1; capillary temperature, 320°C; S lens RF level, 35; heater temperature, 400°C. Nitrogen produced by a nitrogen generator (Peak Scientific) was employed as both the collision and damping gas. Orbitrap mass calibration was performed once a week to ensure a working mass accuracy of < 5 ppm. Pierce LTQ Velos ESI Positive ion and Pierce LTQ Velos ESI Negative ion calibration solutions from Thermo Fisher Scientific (Rockford, IL, USA) were used to calibrate the mass spectrometer. A mass range of 50–1200 m/z and resolving power of 60,000 FWHM at 400 m/z were used for full scan acquisitions. Data dependent acquisitions with an MS/MS list incorporating precursor ion accurate mass and retention time were used for identification experiments. The precursor ions were isolated in the LTQ at an isolation width of 1 m/z, fragmented in the HCD cell and analysed in the Orbitrap at a mass range of 100–750 m/z and resolving power of 60,000 FWHM at 400 m/z. The fragmentation was completed at 4 different collision energies, NCE–10, 30, 50 and 70%. The chromatographic column used was an Acquity BEH C18, 1.7 um 2.1 x 100 mm (Waters, Wexford Ireland) and the mobile phases used were 0.1% formic acid in water (A) and 0.1% formic acid in methanol (B). The ESI+ gradient was as follows, (time in minutes, %B): (0, 1), (2.5, 1), (16, 99), (18, 99), (18.11, 1), (20, 1) with a flow rate of 0.4 ml/min. ESI- employed the same gradient with a flow rate of 0.36 ml/min.

### Data analysis

Xcalibur 2.2 from Thermo Fisher Scientific (MA, USA) was used for instrument control and acquisition of the high resolution LC-MS data. The acquired .raw files were converted to .mzML format using the Proteowizard msconvert tool [15]. The data were uploaded to XCMS online for data pre-processing and analysis. The pre-processing parameters used are as follows: feature detection with centWave (m/z tolerance of 5 ppm, minimum and maximum peak widths of 5 and 20 respectively), retention time correction with obiwarp method (profStep1), chromatographic alignment with mzwid—0.05, minfrac 0.5 and bw 5. The multi-group data analysis was undertaken using the Kruskal-Wallis non-parametric test with post hoc analysis [16]. Peak intensities were normalized against cell counts and column plots for statistically significant metabolites were plotted. The top 200 features were selected from the variable importance in projection (VIP) plots based on their importance to each respective model, which were further short-listed by cross checking their chromatographic peak shapes, peak intensities and p-values from their corresponding spectral profiles in XCMS Online. Significant features with p-value ≤ 0.05, q-value ≤ 0.05 and peak intensity ≥ 10^5^ were chosen for identification. The short-listed ions were fragmented (MS/MS) and metabolite identifications were confirmed by comparing with standards in the high resolution mass spectral library, mzCloud (https://www.mzcloud.org). Multivariate analysis (orthogonal projection to partial least squares discriminant analysis; OPLS-DA) was completed in SIMCA v14.1 (Umetrics, Umea, Sweden) to visualize differences between sample groups and to produce the prediction models. The identified features and their peak areas were analysed using the MSEA tool, which is similar to the gene set enrichment analysis (GSEA), through the MetaboAnalyst 3.0 software [11, 17], where a biologically meaningful pattern was estimated for our identified metabolites using a pre-defined set of metabolites, associated to the metabolic pathways. Peak area data (S1 Table) was uploaded pairwise, as conditions of control (RM) vs. VEGF, PC-3S or PC-3M co-cultured HUVECs. In this case a quantitative enrichment analysis (QEA) was carried out with the peak area data, associated with the identified metabolites, uploaded into the web-based software and a Q-statistic was estimated for each metabolite set that describes the correlation between the peak area profiles, X, of the matched metabolite set and the phenotype labels, Y, which in our case are the different conditions of HUVECs with RM, VEGF, PC-3S and PC-3M cells. Fold enrichment obtained from the MetaboAnalyst application of MSEA is calculated as the ratio of calculated statistic/expected statistic [18, 19]. Statistical significance for the bar graphs were obtained by calculating p-values, represented by: * p ≤ 0.05, ** p ≤ 0.01, *** p ≤ 0.001, **** p ≤ 0.0001.

## Results

We report differential responses of ECs to heterogeneous tumour cell subpopulations, using HUVECs which were co-cultured with prostate cancer cell sub-populations exhibiting extreme metastatic abilities. For this purpose we have used a dual model cell lines derived from the PC-3 cells [12], in which the PC-3M is enriched with epithelial cancer stem cell (CSC) properties, while the PC-3S model lacks this feature (non-CSC) but displays a stable epithelial mesenchymal transition (EMT). We have done an extensive characterization of the CSC features of PC-3M cell line model [12], substantiated by its expression of genetic markers characteristic of stem cells [20, 21]. The completely disconnected properties of CSC and EMT in these cell lines, regardless of their tissue of origin, provides us with ideal models for studying their metabolic influences on ECs based on their distinct metastatic abilities.

**Fig 1:**
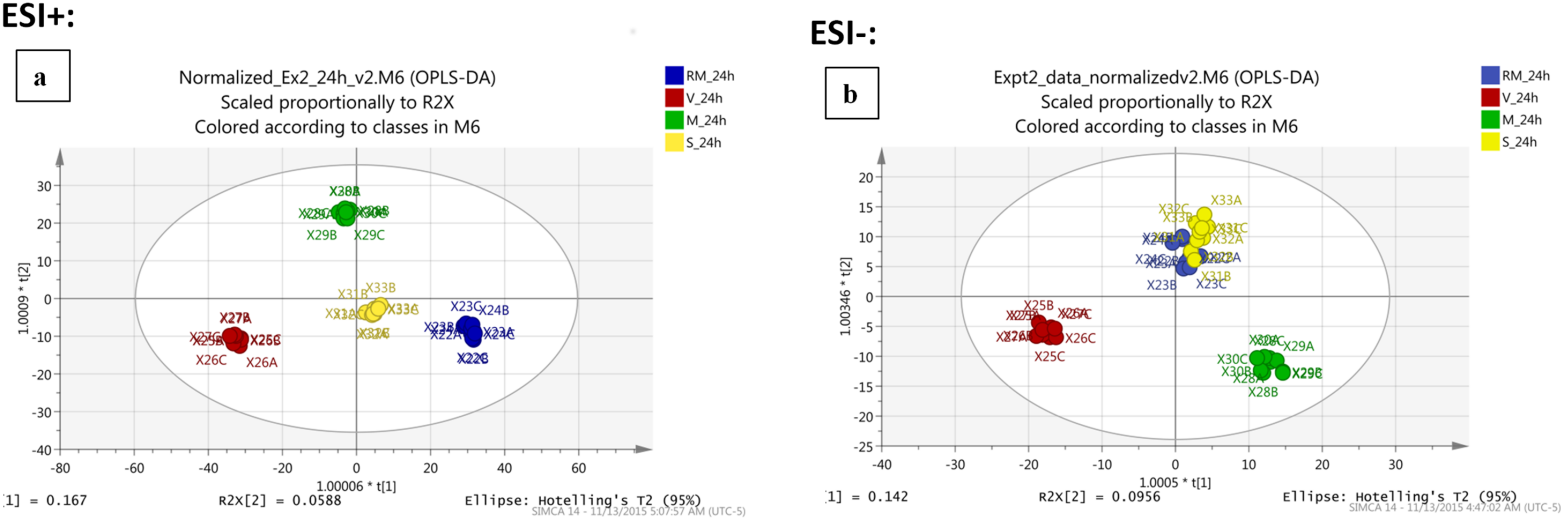
Orthogonal projection to partial least squares discriminant analysis (OPLS-DA) plots: (a) ESI+ ionization mode, (b) ESI- ionization mode. RM_24h –Control HUVECs incubated in restricted medium for 24h, V_24h—HUVECs with VEGF, M_24h –HUVECs co-cultured with PC-3M cells, S_24h –HUVECs co-cultured with PC-3S cells.

The LC/MS data was analysed using XCMS Online which identified 5285 features in ESI+ and 1366 features in ESI- by multi-group analysis, based on retention time and m/z value matches. Multivariate data analysis for all conditions was performed using OPLS-DA (Fig 1). The ESI+ data showed cumulative values of R^2^(Y) = 99.7% and Q^2^ = 86.2% and ESI- showed R^2^(Y) = 97.9% and Q^2^ = 86.3%, where R^2^ indicates the variation shown by all the components in the model and Q^2^ is the accuracy of the model prediction of the class membership. These high values indicate excellent clustering of the groups whilst also demonstrating a clear distinction between control HUVECs and HUVECs submitted to each of the treatment conditions (VEGF, PC-3S cells or PC-3M cells), except PC-3S condition in the ESI- mode.

The detailed fragmentation spectra and the matching spectral profile from the mass spectral library for each of the identified metabolites are shown in S1 Fig. In this study, a quantitative metabolite identification metric was adapted based on the study by Sumner *et*. *al*., 2014 [22], to increase the confidence in identification, for which a minimum identification point (IP) of 5 is suggested. In this work the following features were considered to calculate the IP score: accurate mass match with a tolerance of 5 ppm (1.0 IP), accurate mass tandem mass spectrum (2.0 IP), and molecular formula from accurate mass and isotopic pattern (1.0 IP). The total score for each metabolite is calculated as, (1+2+1)*1.5 = 6 (the score is multiplied by 1.5 for spectral library match or 2 for the use of standards). Thus by performing fragmentation of the short-listed ions and comparing the resulting spectra with the mass spectral library a list of the putatively identified metabolites were generated, that are displayed in Table 1. It can be observed that, while the ESI+ mode can detect higher number of features and metabolites, both ESI+ and ESI- modes of acquisition provide complementary information and different sets of metabolites, except for the redundancy observed in L-glutamic acid, pantothenic acid and L-glutathione (normalized peak areas are shown in S1 Table).

**Table 1:** Putative metabolite identifications with ESI+ and ESI- modes.

**ESI+**			
**Identified metabolites**	**Accurate mass from XCMS online [M+H]**	**Retention time (min)**	**p-value**
L-Glutamic acid	148.0600	0.70	0.00509
L-Arginine	175.1182	0.65	0.00007
L-Glutathione reduced	308.0900	1.15	0.00067
L-Tryptophan	205.0965	5.30	0.00001
L-Tyrosine	182.0805	1.49	0.00002
Methionine	150.0579	1.13	6.07226e-6
Nicotinamide dinucleotide (NAD)	664.1141	1.30	7.25358e-6
Pantothenic acid	220.1173	5.09	0.00004
Oleamide	282.2781	16.32	0.00342
γ-L-Glutamyl-L-Glutamic acid	277.1021	0.93	0.00125
Inosine	269.0873	2.71	0.00291
Spermine	203.2223	0.51	ns
*trans* 3-Indole acrylic acid/Indole 3-acrylic acid	188.0700	5.30	0.00001
Cysteinylglycine	179.0479	1.15	0.00046
Guanine	152.0561	0.91	0.00017
Creatine	132.0763	0.73	0.0005
Hypoxanthine*	137.0453	1.19/2.71	0.00007/0.00401
Acetyl L-Carnitine (ALC)	204.1222	1.01	1.66321e-6
**ESI-**			
**Identified metabolites**	**Accurate mass from XCMS online [M-H]**	**Retention time (min)**	**p-value**
L-Aspartate	132.0298	0.74	0.01284
L-Glutamic acid^#^	146.0454	0.75	ns
Glycerol 3-phosphate	171.0058	0.82	ns
Pantothenic acid^#^	218.1026	5.26	0.00013
Uridine	243.0613	1.72	0.00445
L-Glutathione reduced^#^	306.0752	1.24	0.00052
Uridine monophosphate	323.0273	1.10	0.04184
Adenosine monophosphate	346.0544	1.11	ns
Galactonic acid	195.0503	0.76	ns
Guanosine	282.0834	2.82	0.01496

*Hypoxanthine shows two isotopic peaks at the same accurate mass.

^#^Metabolites present both in ESI+ and ESI- modes. p-values extracted from the XCMS Online data analysis are for the entire group (Kruskal-Wallis non-parametric statistical test).

ns—not significant.

**Fig 2:**
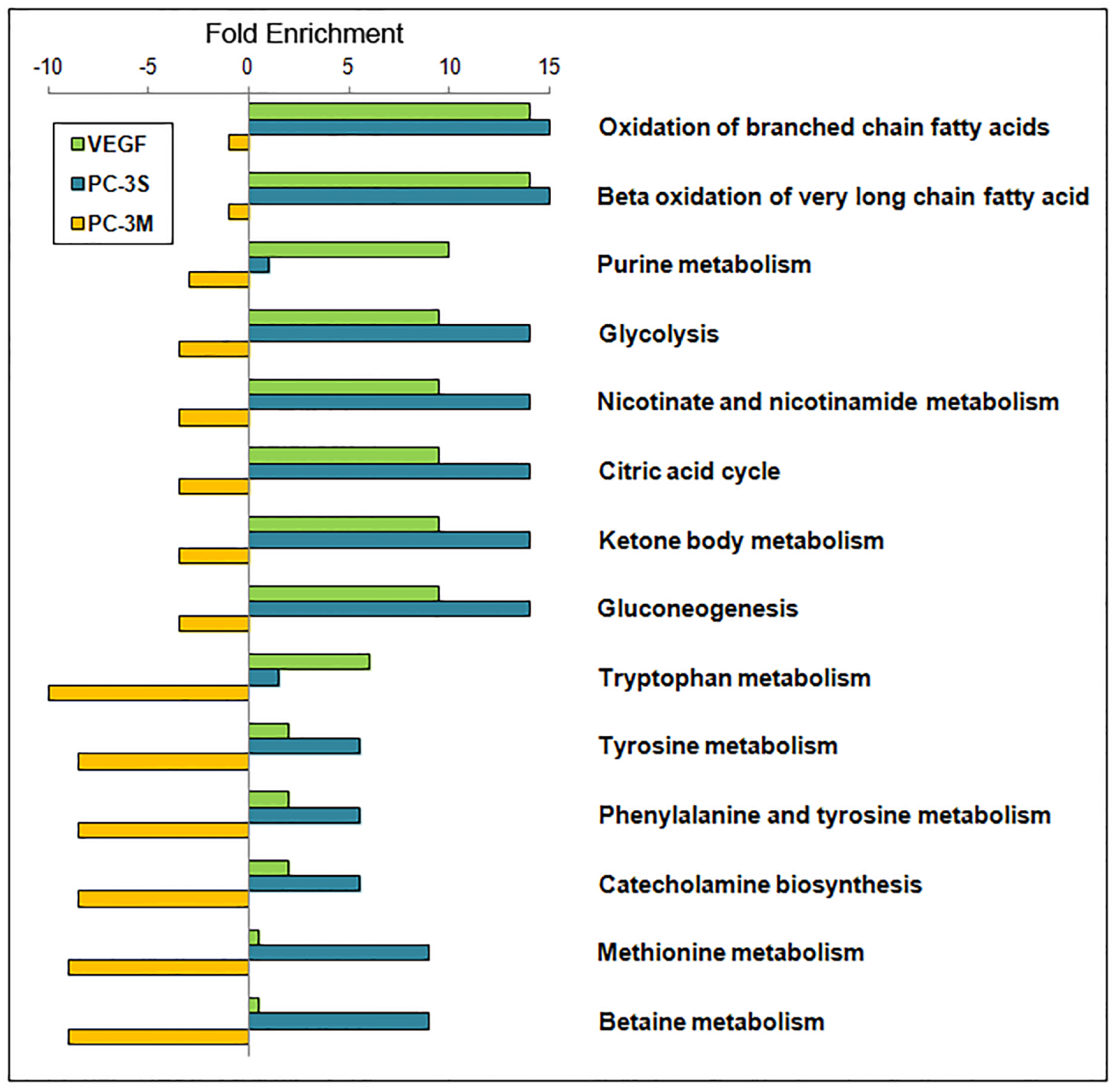
Pathways associated with metabolite set enrichment analysis on HUVEC in the presence of VEGF, PC-3S and PC-3M cells compared to control. This representation shows the pathways enriched by the identified metabolites, irrespective of the upregulation or downregulation of the individual metabolites within the pathways identified. This illustration is adopted from the MSEA considering only the pathways associated with metabolites of higher fold enrichment, whose complete representation is shown in S2 Fig.

The quantitative MSEA [18] of the peak area profiles (S1 Table) revealed that the majority of the metabolic pathways that are significantly altered in HUVECs in response to VEGF are shared with variations observed upon co-culture with PC-3S cells (Fig 2). These include pathways related to fatty acid β-oxidation (associated with acetyl L-carnitine, ALC) and glycolysis, nicotinate and nicotinamide metabolism, citric acid cycle, ketone body metabolism and gluconeogenesis (all of which are related to nicotinamide dinucleotide, NAD). Not all metabolic pathways were equally altered in both conditions. Thus, VEGF, but not PC-3S co-culture, induced a significant change in purine metabolism (hypoxanthine, guanine and adenosine monophosphate (AMP)), while co-culture with PC-3S cells, but not VEGF, significantly altered glutathione, pantothenate and amino acid metabolism.

Contrastingly, HUVECs co-cultured with the highly metastatic PC-3M cells exhibited a metabolic profile completely distinct from that observed in response to VEGF or co-culture with PC-3S cells (Fig 2). The tryptophan, phenylalanine, tyrosine, catecholamine, methionine and betaine metabolic pathways were found to be significantly affected, while none of the major pathways affected in HUVECs by VEGF stimulation and/or PC-3S co-culture were significantly affected by PC-3M co-culture (Fig 2).

**Fig 3:**
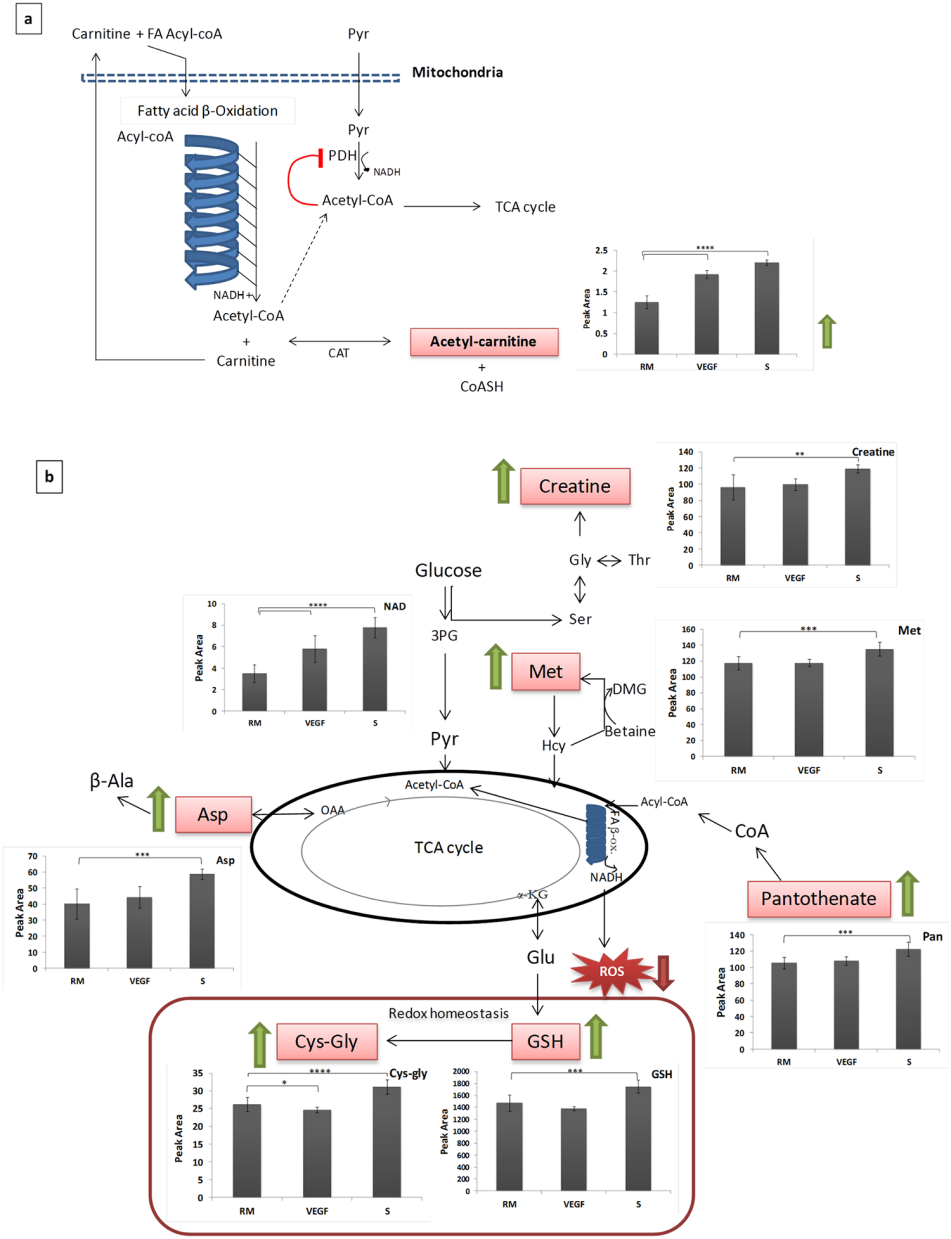
Metabolite changes due to VEGF and PC-3S cells on HUVECs, incorporated into metabolic pathways identified by MSEA. (a) Upregulation of ALC, associated to β-oxidation of fatty acids, (b) NAD-induced pathway alterations and other alterations only induced by PC-3S cells. 3PG– 3-phoshogluconate, αKG– α-ketoglutarate, β-Ala– β-Alanine, ALC—acetyl L-carnitine, Asp—aspartate, CAT—Carnitine acetyltransferase, CoASH—reduced co-enzyme A, Cys-Gly—cysteinylglycine, DMG—Dimethylglycine, FA Acyl-Coa—fatty acid acyl-CoA, Hcy—homocysteine, Gly—glycine, GSH—glutathione reduced, Glu—glutamate, Met—methionine, OAA—oxaloacetate, PDH—pyruvate dehydrogenase, Pyr—pyruvate, ROS—reactive oxygen species, Ser—serine, Thr—threonine, RM—control HUVECs, VEGF—HUVECs treated with VEGF, S—HUVECs co-cultured with PC-3S. Peak area = Peak area×10^5^ (A.U. per 10^6^ cells).

Although the MSEA-based metabolite pathway enrichment arranges the identified metabolites into meaningful metabolic pathways, it does not provide information about the direction (upregulation or downregulation) of the enrichment. In order to complement this analysis, bar graph models were constructed for the peak areas of the identified metabolites, incorporating them into the pathways shown as altered in MSEA. These models show that ALC was upregulated in HUVECs both under VEGF stimulus and PC-3S co-culture (Fig 3a), with ≥ 1.5 fold-change in both cases, associated with fatty acid oxidation, as inferred from MSEA (Fig 2). They also show an upregulation of NAD, creatine, methionine, pantothenate, reduced glutathione, cysteinylglycine and aspartate (Fig 3b), again consistent with the pathways identified by MSEA (Fig 2). Interestingly, while NAD was upregulated in both VEGF and PC-3S conditions (1.6X and 2.2X, respectively), all other metabolites were significantly upregulated only upon co-culture with PC-3S cells, but not upon exposure to VEGF, although at lower fold changes.

**Fig 4:**
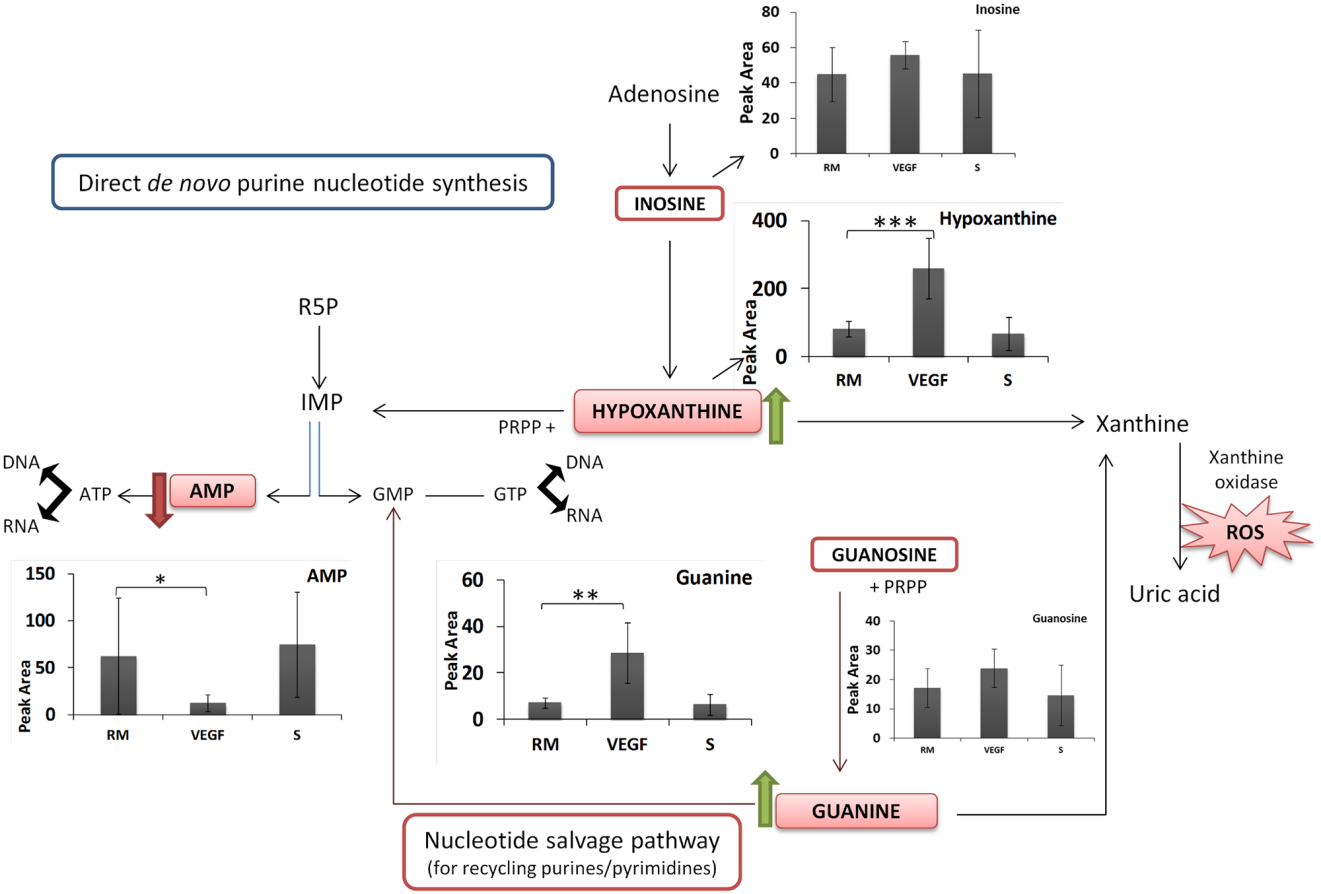
Metabolite changes associated with purine metabolism altered with VEGF but not observed with PC-3S. The graphs show an upregulation of hypoxanthine and guanine and downregulation of AMP. AMP—adenine monophosphate, ATP—adenine triphosphate, GMP—guanine monophosphate, GTP—guanine triphosphate, IMP—inosine monophosphate, PRPP—Phosphoribosyl pyrophosphate, R5P –ribose-5-phosphate, ROS—reactive oxygen species, RM—control HUVECs, VEGF—HUVECs treated with VEGF, S—HUVECs co-cultured with PC-3S. Peak area = Peak area×10^5^ (A.U. per 10^6^ cells).

Hypoxanthine, guanine and AMP, were significantly altered in HUVECs exposed to VEGF, but not co-cultured with PC-3S (Fig 4), with an upregulation of > 3X in the case of first two metabolites and a downregulation of ~5X in AMP. This is also consistent with the MSEA enrichment of the purine metabolism pathway in HUVECs treated with VEGF but not co-cultured with PC-3 cells (Fig 2). The similarity in metabolic changes induced by VEGF and PC-3S co-culture may be partly explained by the expression of VEGF-A by PC-3S cells (S3 Fig shows the upregulation of VEGF-A gene expression by PC-3S cells to that of PC-3M cells).

**Fig 5:**
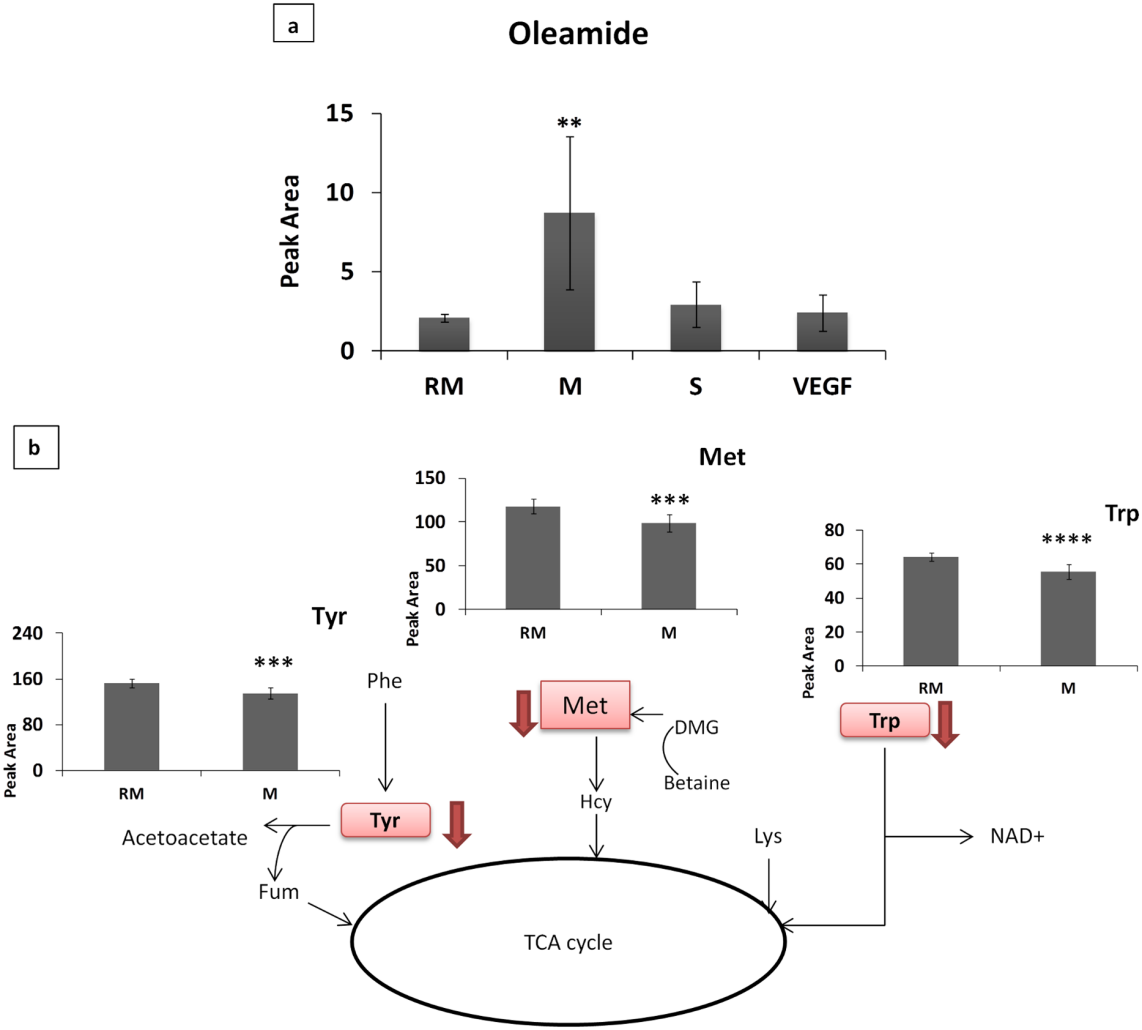
Metabolite changes and pathway enrichments identified by MSEA in RM and PC-3M-HUVEC pair. Peak area changes of: (a) oleamide in all the conditions, and (b) tyrosine, methionine and tryptophan by PC-3M condition. DMG—Dimethylglycine, Fum—fumarate, Hcy—homocysteine, Lys—lysine, Met—Methionine, Phe—phenylalanine, Trp—Tryptophan, Tyr—Tyrosine, RM—control HUVECs, VEGF—HUVECs treated with VEGF, S—HUVECs co-cultured with PC-3S cells, M—HUVECs co-cultured with PC-3M cells. Peak area = Peak area×10^5^ (A.U. per 10^6^ cells).

This analysis also showed that the metabolite undergoing the most significant and specific changes in HUVECs co-cultured with PC-3M cells was oleamide, with a 4X upregulation (Fig 5). This metabolite is not associated with any of the pathways inferred from MSEA as significantly altered in HUVECs co-cultured with PC-3M cells (Fig 2). In addition, tryptophan, tyrosine and methionine showed a downregulation in HUVECs co-cultured with PC-3M cells, which was not observed in the other conditions (Figs 2 and 5). These observations strongly suggest that the response of ECs to epithelial CSCs, represented by PC-3M cells in our model, is strikingly distinct from responses to mesenchymal non-CSCs, represented by PC-3S cells, or to the canonical angiogenic factor, VEGF.

## Discussion

In order to grow and spread to other organs, cancer cells recruit ECs for tumour angiogenesis [23] and to achieve this they induce several changes in ECs that can alter their molecular signalling and metabolic pathways. Studies on molecular angiogenesis have led to the development of anti-angiogenic therapies in previous years, although the success rate has been low [24]. Although EC metabolism has been under study in the past few years they have not been particularly focused on the direct effect of cancer cells on the ECs [25]. Specific tumour subniches, including those sustaining CSC, contribute to the development of vascular niche and tumour-associated angiogenesis [26, 27]. However, whether tumour cell subpopulations with distinct CSC or non-CSC features differentially affect endothelial cell phenotypes and metabolism have not been systematically approached. We have addressed this issue by co-culturing HUVECs with clonal tumour cell sublines with stable and clearly distinct CSC or non-CSC properties [12].

In the present study, the application of high resolution mass spectrometry has yielded a global metabolic fingerprint of the ECs exposed to diffusible factors produced by these distinct cell subpopulations or to VEGF. We report that the most significantly altered metabolic changes induced by non-metastatic and strongly mesenchymal-like PC-3S cells are similar to those induced by VEGF, which includes metabolites such as ALC and NAD. These similarities may be partly explained by the observation that PC-3S cells express VEGF-A at levels significantly higher than PC-3M cells. Similarly, it has been found *in vivo* that the low metastatic DU145 cell line secretes VEGF at significantly higher levels than PC-3M cells [28].

One of the most significant changes observed in HUVECs common to VEGF treatment and PC-3S co-culture was the upregulation of ALC. This metabolite is produced from carnitine and acetyl-coA by carnitine acetyltransferase (CAT) and thus is closely associated to fatty acid oxidation, which generates acetyl-CoA. This reaction is reversible and carnitine produced from the breakdown of ALC can be recycled back into the cytoplasm to transport more fatty acid acyl-coA’s for β-oxidation [29], which in turn produces more acetyl-CoA. Fatty acid oxidation has been found to be important in ECs mainly due to its contribution to dNTP synthesis and driving endothelial cell proliferation for vessel sprouting [30]. Further, ALC has been reported to possess therapeutic implications in protecting vascular function against oxidative stress [31] and in protecting endothelial structure in blood-brain barrier [32].

A second metabolite strongly upregulated in HUVECs by both VEGF and PC-3S cells was NAD, a major contributor to a variety of metabolic pathways (glycolysis, nicotinamide metabolism, citric acid cycle, ketone body metabolism and gluconeogenesis). Glycolysis has been found to be important for endothelial cell survival, proliferation and other angiogenic activities such as migration and sprouting [33, 34]. Endothelial mitochondrial function is not reported to be a significant or preferred pathway for ATP production, compared to glycolysis, although it has been reported to be useful during cellular stress or when glycolysis is compromised [35, 36], in addition to the angiogenesis stimulation by non-toxic levels of mitochondrial ROS [37, 38]. The interpretation of these observations require a certain degree of caution, given that related reactions involve inter-conversions between the oxidized and the reduced forms of NAD. The reduced NADH form is unstable and hence the observed NAD levels are likely to reflect a mixture of both forms, which makes NAD a relatively unreliable biomarker.

Importantly, PC-3S cells induced the upregulation of metabolites not significantly affected by VEGF, while VEGF, but not PC-3S cells, strongly altered purine metabolism. These differences between the two conditions are likely explained by the production by PC-3S cells of diffusible factors other than VEGF, yet to be identified. VEGF is a potent regulator of endothelial cell proliferation and angiogenesis [39] and in order to proliferate, cells have to generate an excess of nucleotide components. Purine metabolism is important for the production of DNA and RNA components. Moreover, they can also generate xanthine oxidase-derived ROS. In contrast, it has been shown that VEGF can induce the production of mitochondrial ROS which in turn can function as signalling factors for mediating endothelial cell migration [40]. ROS-mediated angiogenesis is also observed by NAD(P)H-generated ROS [41]. To our knowledge, the changes in hypoxanthine, guanine and AMP induced by VEGF in ECs have not been reported previously. Hence, the changes in purine metabolism induced by VEGF are explained as associated with a concomitant induction of endothelial cellular proliferation and may also be related to the generation of ROS.

The most remarkable finding reported herein is the extremely contrasting metabolic response of HUVECs to two tumour cell subpopulations displaying distinct phenotypes. Thus, while the non-metastatic PC-3S cells induced metabolic changes in HUVECs that were largely similar to those induced by VEGF, the highly metastatic PC-3M cells, enriched in CSCs, induced a completely distinct set of metabolic alterations. These included a strong upregulation of oleamide and a significant, but of lower fold change, downregulation of tryptophan, tyrosine and methionine.

Oleamide is a primary fatty acid amide, reported to possess signalling [42] and sleep-inducing properties [43] and has vasodilatory effects [44, 45]. In addition it is found to regulate cellular gap junctions in various types of cells [46–49]. The gap junction inhibition, when oleamide is supplemented as a therapeutic agent, leads to an anti-metastatic effect by inhibiting the connexin proteins that enable gap junction-mediated intercellular communications during metastasis [50, 51]. Furthermore, oleamides are suspected to play a role in preventing the spread of apoptotic proteins or cell death signals from a damaged cell to a neighbour through cellular junctions [52, 53]. Endothelial cell damage and apoptosis have been found to be induced by tumour cells during metastasis, in order to extravasate from the blood vessels to invade secondary metastatic sites [54, 55]. Thus, the intracellular production of oleamide shows an endothelial response to the metastatic potential of PC-3M cells. Although oleamide has been reported in a variety of biological matrices to include human breast cancer cells [56], mouse neuroblastoma cells [57], human serum [58] and rat cerebral spinal fluid [59], it is yet to be reported in ECs.

PC-3M cells are also reported to be metabolically different from the PC-3S sub-type, in which the PC-3M cells depend mainly on aerobic glycolysis and use mitochondrial substrates as alternative energy sources, while the PC-3S cells rely on mitochondrial oxidation for energy [60]. Hence PC-3M cells produce high amounts of lactate compared to PC-3S cells [60]. ECs reportedly take up lactate which functions as signalling molecule for HIF-1 activation, consecutively increasing endothelial cell migration, tube formation and angiogenesis [61, 62]. Thus the unique properties of the two PC3 sub-types explain their distinct metabolic shifts in HUVECs.

A tumour mass can have different kinds of tumour cells co-existing within the vascular niche [63, 64] and the heterogeneous tumour sub-types with EMT and CSC traits have been reported to initiate and promote angiogenesis by the production of VEGF [65]. Further, it has also been reported that the dormant niche and the micrometastatic outgrowths are promoted by different kinds of vasculature systems, where the ECs within the stable microvasculature secrete thrombospondin-1 that promotes the dormancy of the tumour cells, while the sprouting neovasculature secretes periostin and transforming growth factor-β1 (TGF-β1) favouring proliferation of the metastatic tumour cells [66]. This shows that a vascular heterogeneity exists within tumour subtypes. In our study the strikingly different metabolic changes observed within the ECs grown with EMT- and CSC-like prostate cancer cells strongly support differing behaviour of the vasculatures within the tumour subtypes.

## Conclusion

In this study, we have discovered that the ECs reprogram their metabolism by displaying unique metabolic responses to diffusible factors produced either by a CSC-enriched tumour cell subpopulation or by a non-CSC tumour cell subpopulation. To our knowledge, this is the first time that tumour heterogeneity has been reported to influence endothelial cell metabolism, and thus presumably functions, through distinct and largely non-overlapping responses either to CSC or to non-CSCs. Our approaches, which include the use of adequate cell models, lay the foundations for an improved understanding of tumour-associated angiogenesis that takes into account distinct tumour cell subpopulations and their specific interactions with, and modulation of, endothelial cells.

## Supporting information

S1 Table Quantitative values of the peak areas of the identified metabolites, in ESI+ and ESI- modes. (XLSX)

S1 Fig Fragmentation spectra and the matching spectral profile from the mass spectral library for the identified metabolites. (PDF)

S2 Fig Pathways generated with identified metabolites in HUVEC in the presence of VEGF, PC-3/S and PC-3/M cells, by metabolite set enrichment analysis using MetaboAnalyst 3.0. (DOCX)

S3 Fig The fold change ratio of the VEGF-A gene expression data, showing VEGF-A expressed by PC-3S to PC-3M cells, where PC-3S cells show higher expression relative to PC-3M cells. (DOCX)
