# Supplementary material for: Untargeted metabolomics reveals distinct metabolic reprogramming in endothelial cells co-cultured with CSC and non-CSC prostate cancer cell subpopulations
Source: PLoS One. 2018 Feb 21;13(2):e0192175. doi: 10.1371/journal.pone.0192175 (PMC5821452; doi:10.1371/journal.pone.0192175)
Supplement: S1 Fig — (PDF) [file pone.0192175.s002.pdf]

S1 Fig: Fragmentation spectra and the matching spectral profile from the mass spectral library for the identified metabolites.

(a) Chromatographic peaks from XCMS Online, (b) accurate mass of the precursor ion, (c) fragmentation spectral match of the identified metabolites from mzcloud and (d) the isotopic pattern match. Some metabolites do not show peaks in XCMS online as $p > 0.05$. RM – control HUVECs grown in restricted medium, VEGF – HUVECs grown in restricted medium supplemented with VEGF, M – HUVECs co-cultured with PC-3/M cells, S – HUVECs co-cultured with PC-3/S cells.

Glutamate

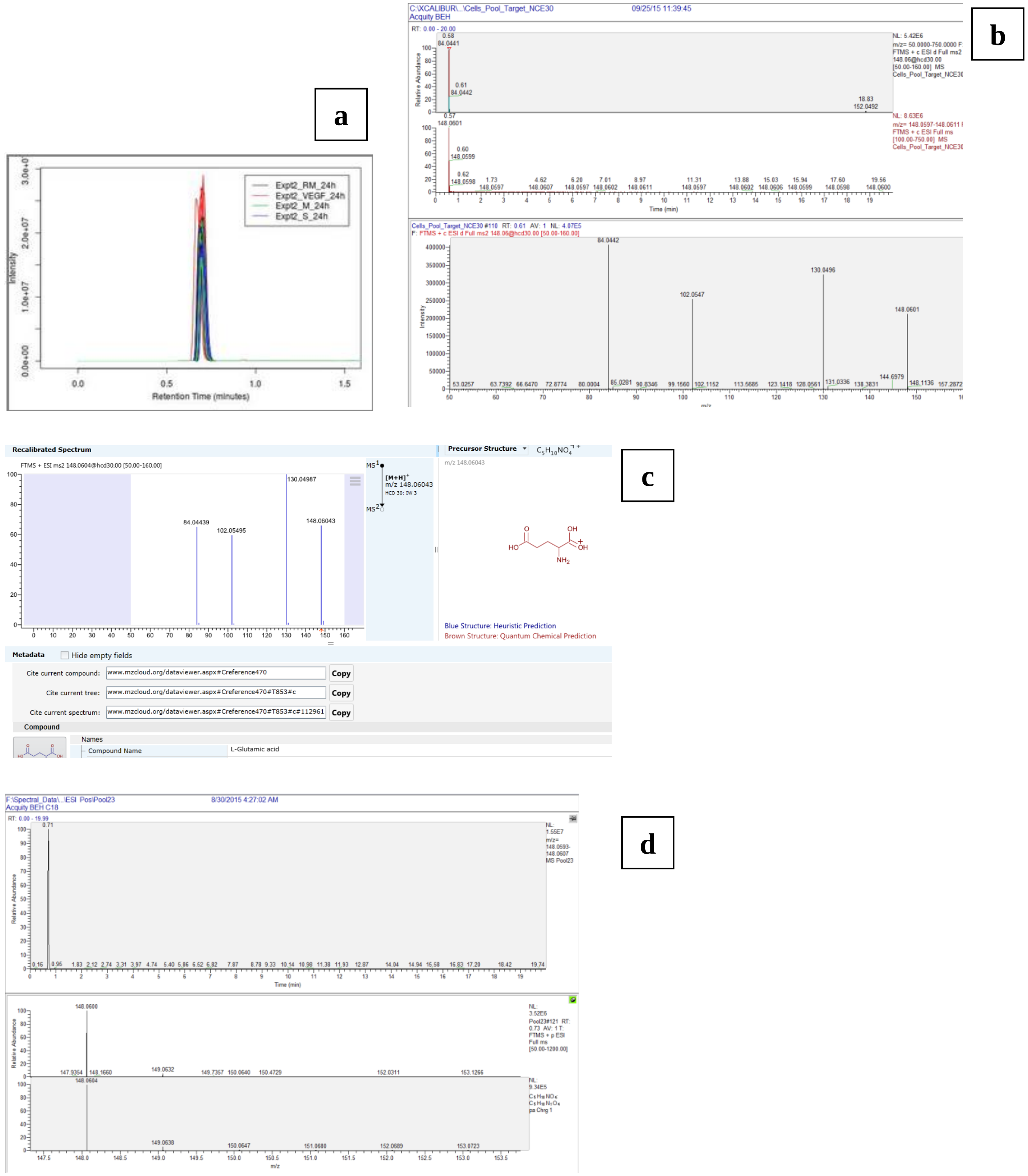
a
Expt2_RM_24h
Expt2_VEGF_24h
Expt2_M_24h
Expt2_S_24h
Intensity
Retention Time (minutes)
b
C:\XCALIBUR\...\Cells_Pool_Target_NCE30
09/25/15 11:39:45
Acquity BEH
Relative Abundance
Time (min)
Cells_Pool_Target_NCE30 #110 RT: 0.61 AV: 1 NL: 4.07E5
F: FTMS + c ESI d Full ms2 148.06@hcd30.00 [50.00-160.00]
84.0442
130.0496
102.0547
148.0601
Intensity
c
Recalibrated Spectrum
FTMS + ESI ms2 148.0604@hcd30.00 [50.00-160.00]
130.04987
148.06043
84.04439
102.05495
[M+H]+
m/z 148.06043
Precursor Structure
m/z 148.06043
Blue Structure: Heuristic Prediction
Brown Structure: Quantum Chemical Prediction
Metadata
Hide empty fields
Cite current compound:
www.mzcloud.org/dataviewer.aspx#Creference470
Copy
Cite current tree:
www.mzcloud.org/dataviewer.aspx#Creference470#T853#c
Copy
Cite current spectrum:
www.mzcloud.org/dataviewer.aspx#Creference470#T853#c#112961
Copy
Compound
Names
Compound Name
L-Glutamic acid
d
F:\Spectral_Data\...\ESI Pos\Pool23
8/30/2015 4:27:02 AM
Acquity BEH C18
Relative Abundance
Time (min)
148.0600
148.0604
m/z

L- Arginine

a

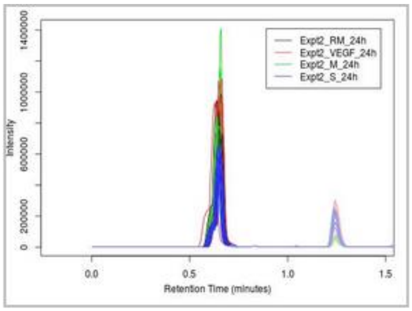

b

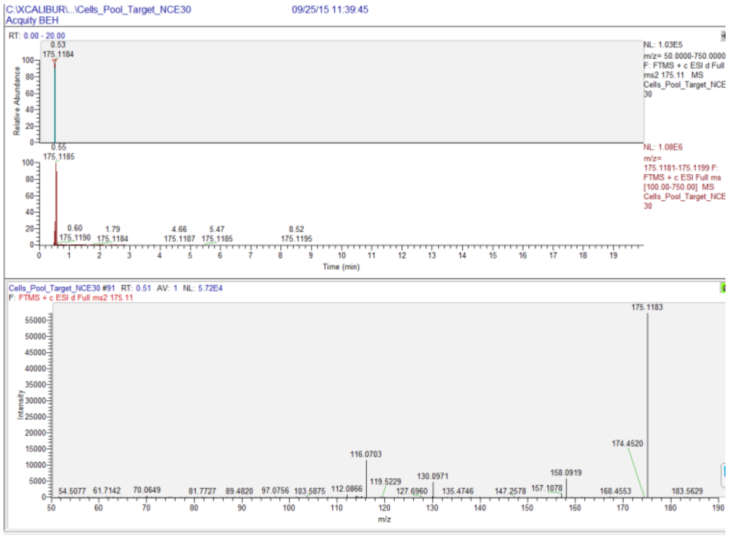

c

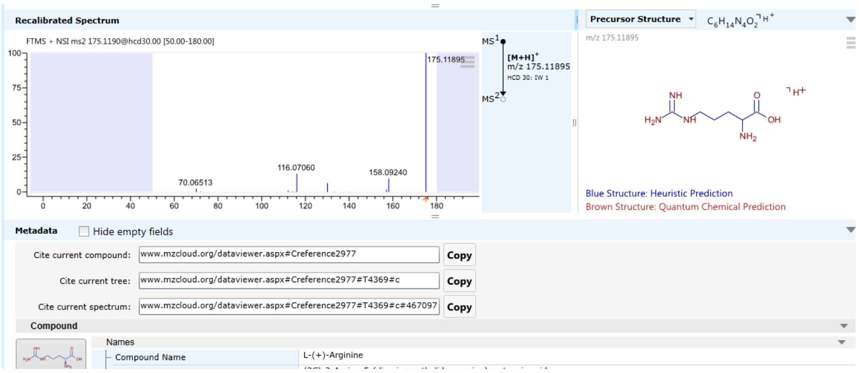

d

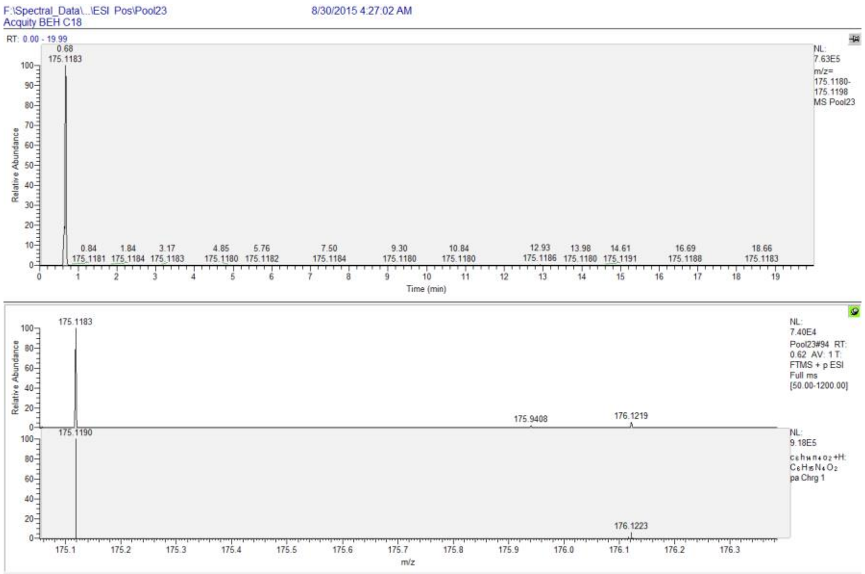

L- Glutathione reduced

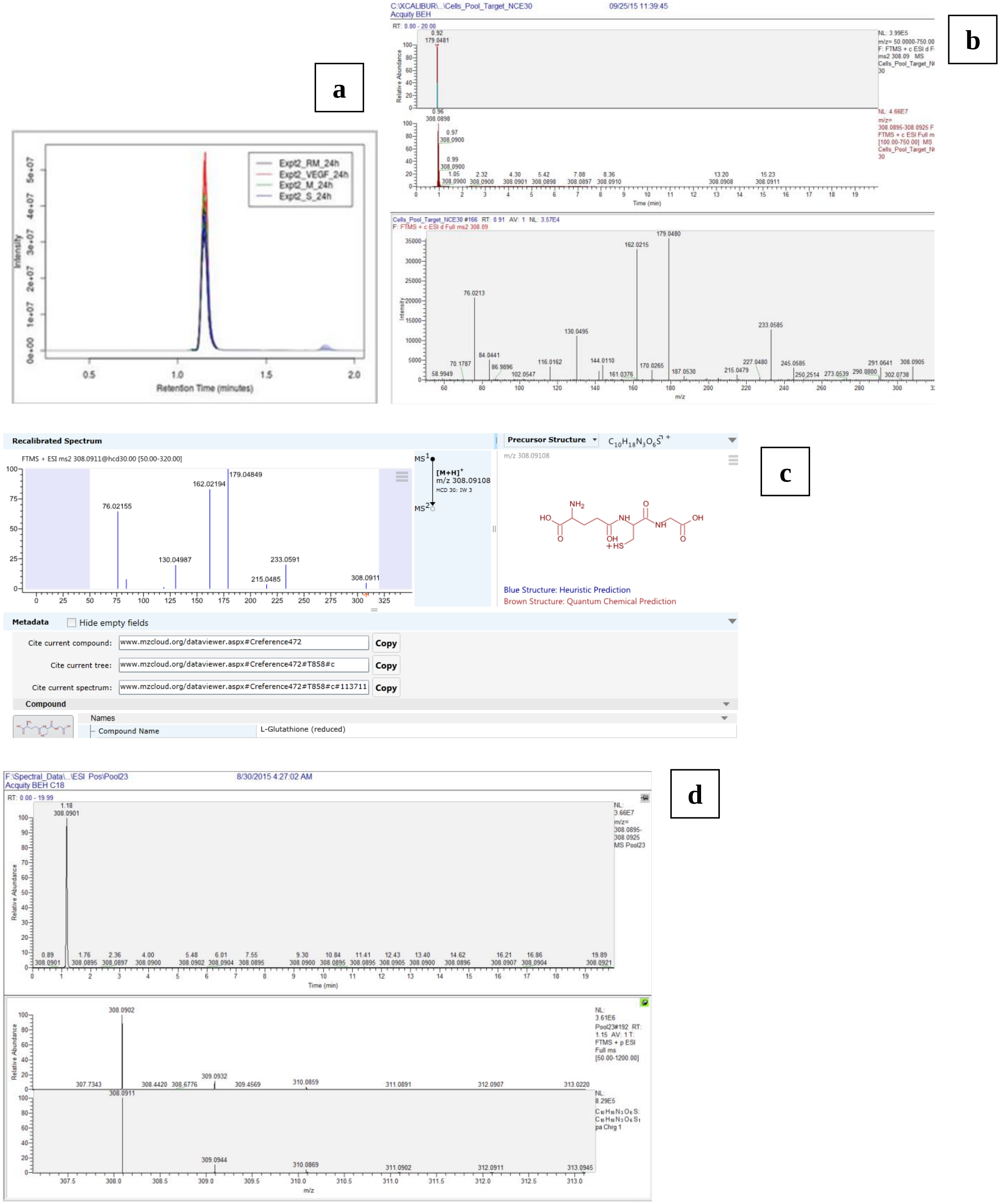

L-Tryptophan

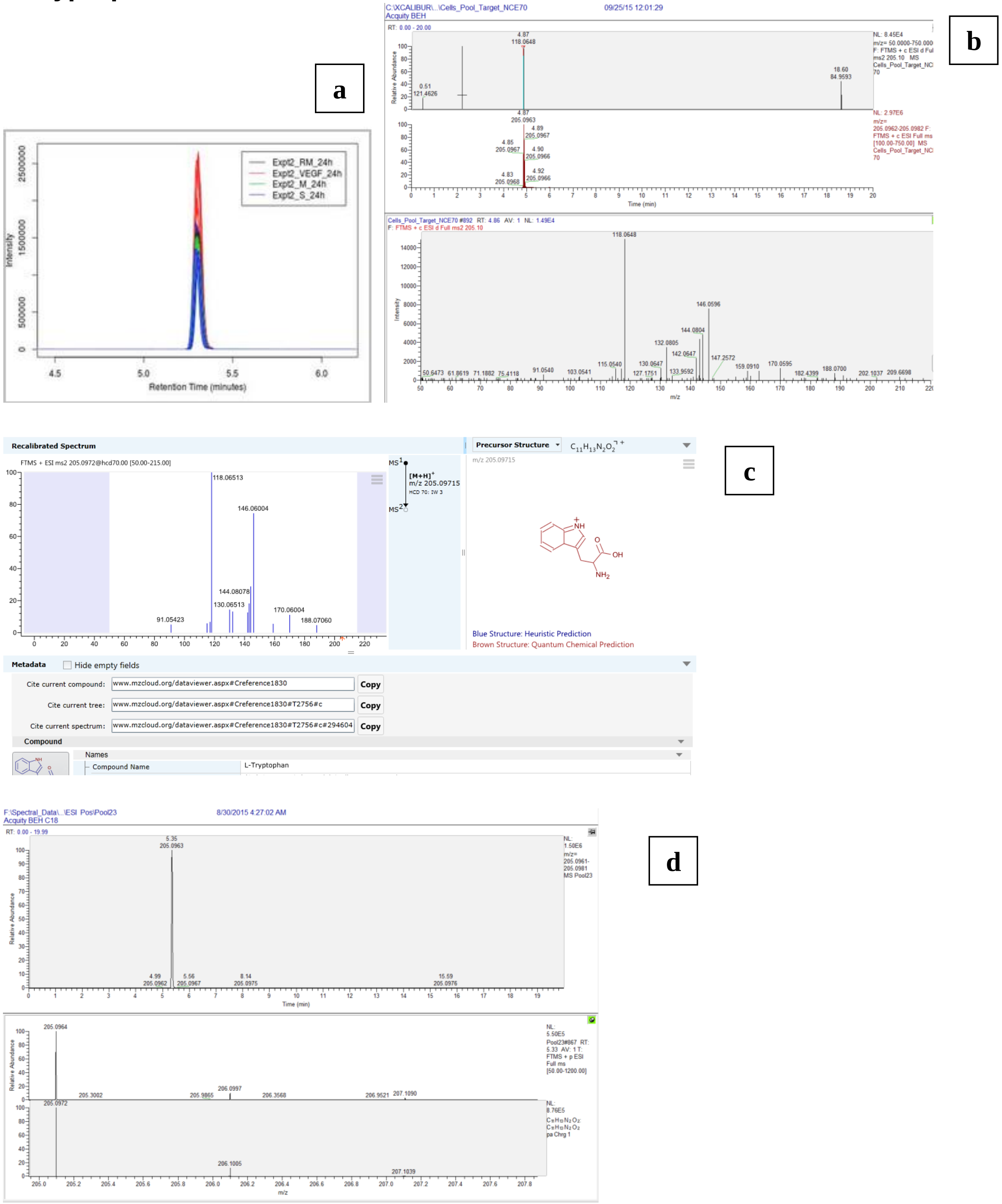

L-Tyrosine

a

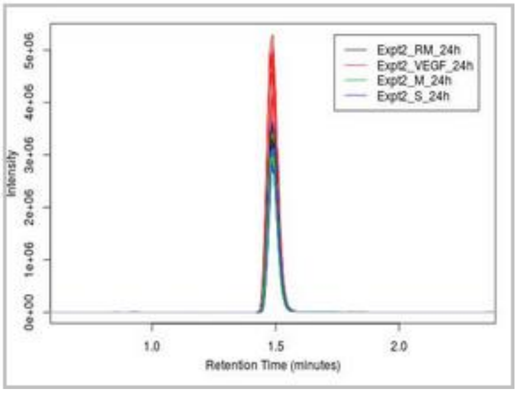

Expt2_RM_24h
Expt2_VEGF_24h
Expt2_M_24h
Expt2_S_24h
Intensity
Retention Time (minutes)

b

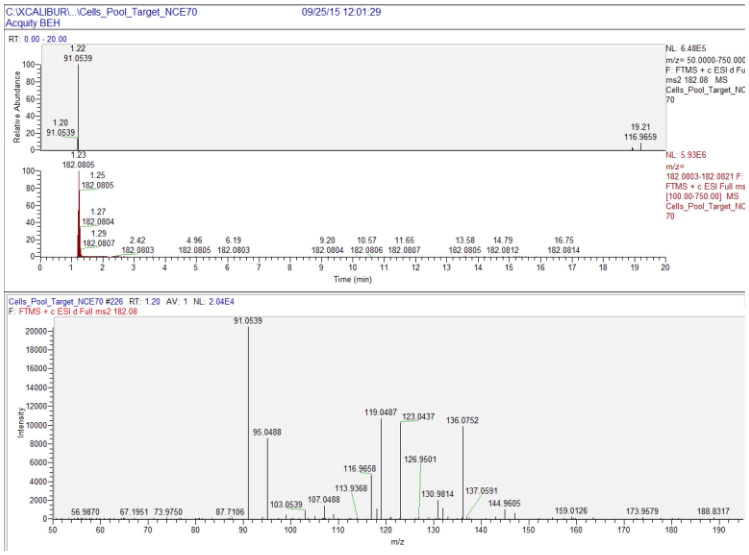

C:\XCALIBUR\...\Cells_Pool_Target_NCE70
09/25/15 12:01:29
Acquity BEH
RT: 0.00 - 20.00
Relative Abundance
Time (min)
Cells_Pool_Target_NCE70 #226 RT: 1.20 AV: 1 NL: 2.04E4
F: FTMS + c ESI d Full ms2 182.08
Intensity
m/z

c

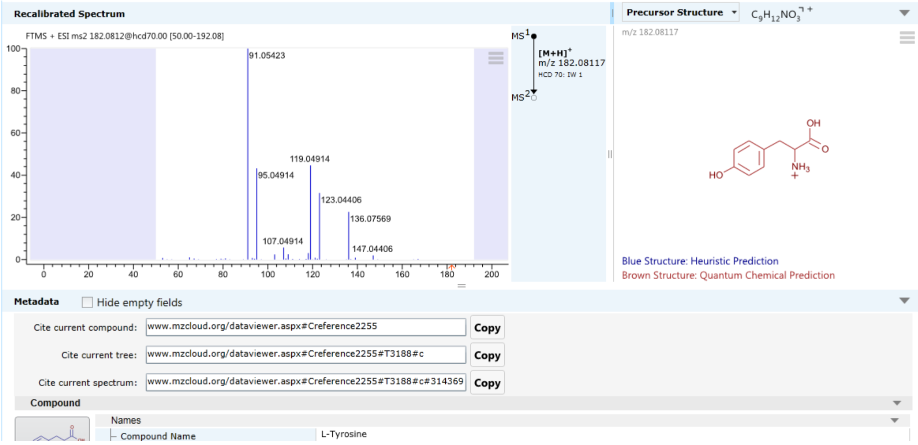

Recalibrated Spectrum
FTMS + ESI ms2 182.0812@hcd70.00 [50.00-192.08]
Precursor Structure
m/z 182.08117
Blue Structure: Heuristic Prediction
Brown Structure: Quantum Chemical Prediction
Metadata
Hide empty fields
Cite current compound:
www.mzcloud.org/dataviewer.aspx#Creference2255
Cite current tree:
www.mzcloud.org/dataviewer.aspx#Creference2255#T3188#c
Cite current spectrum:
www.mzcloud.org/dataviewer.aspx#Creference2255#T3188#c#314369
Copy
Compound
Names
Compound Name
L-Tyrosine

d

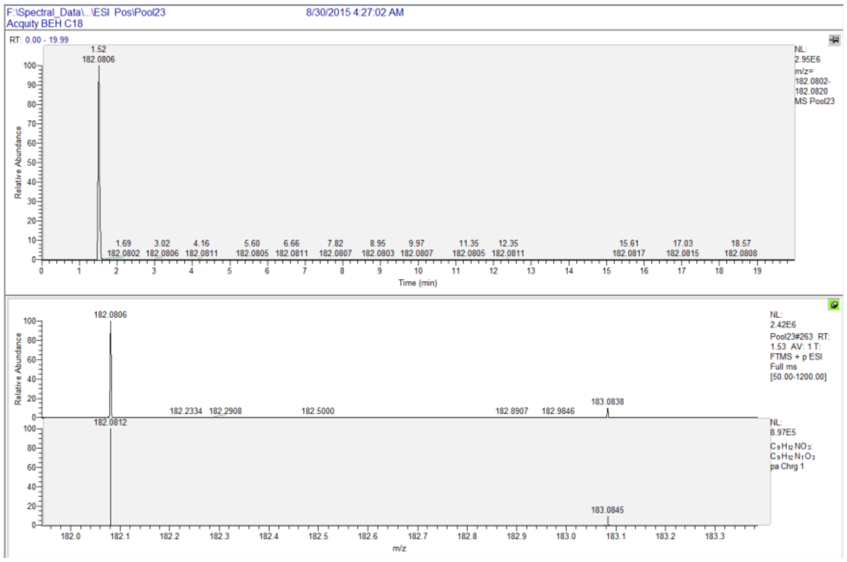

F:\Spectral_Data\...\ESI Pos\Pool23
8/30/2015 4:27:02 AM
Acquity BEH C18
RT: 0.00 - 19.99
Relative Abundance
Time (min)
m/z

Methionine

a

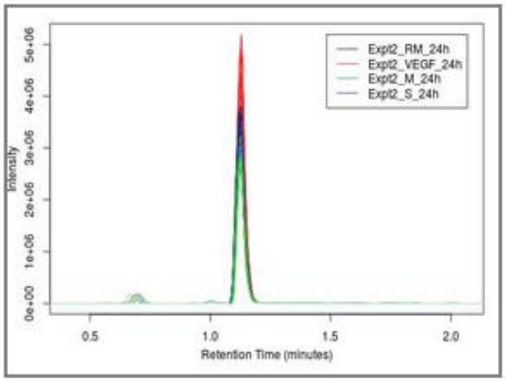

b

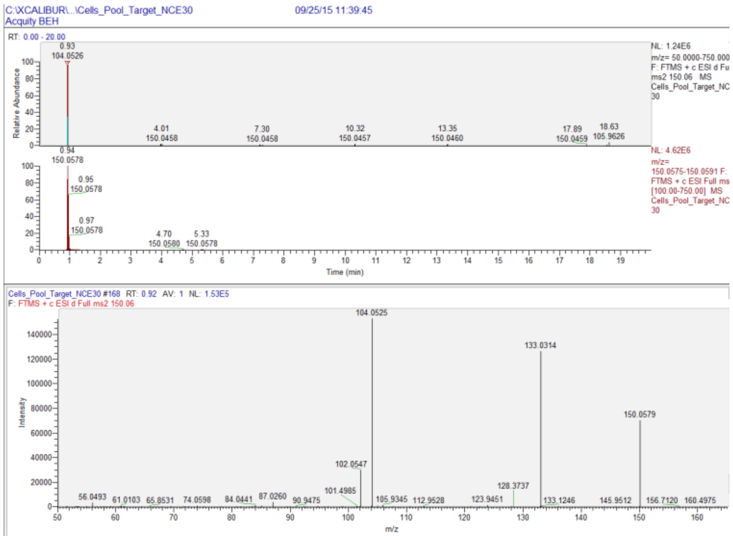

c

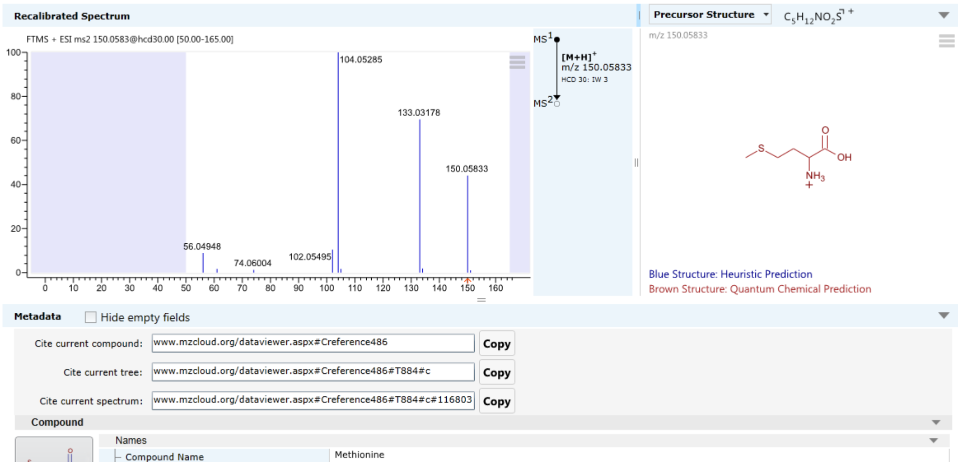

d

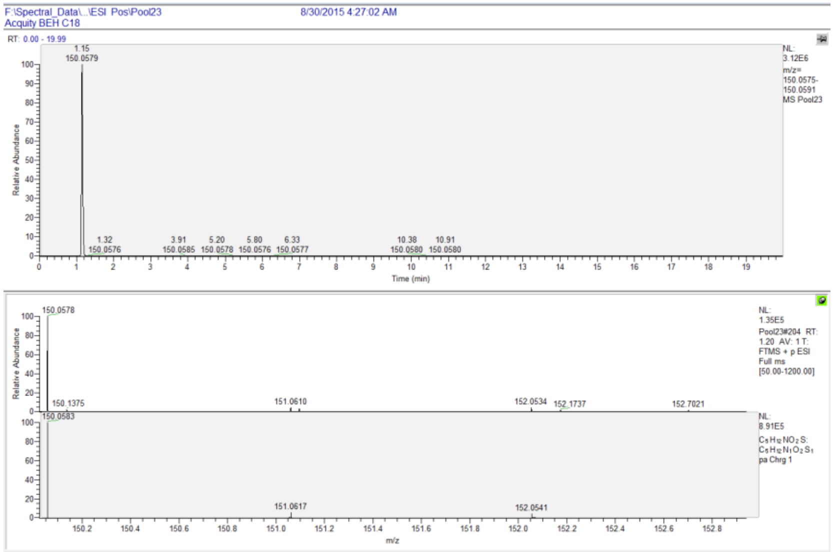

Nicotinamide dinucleotide (NAD+)

b

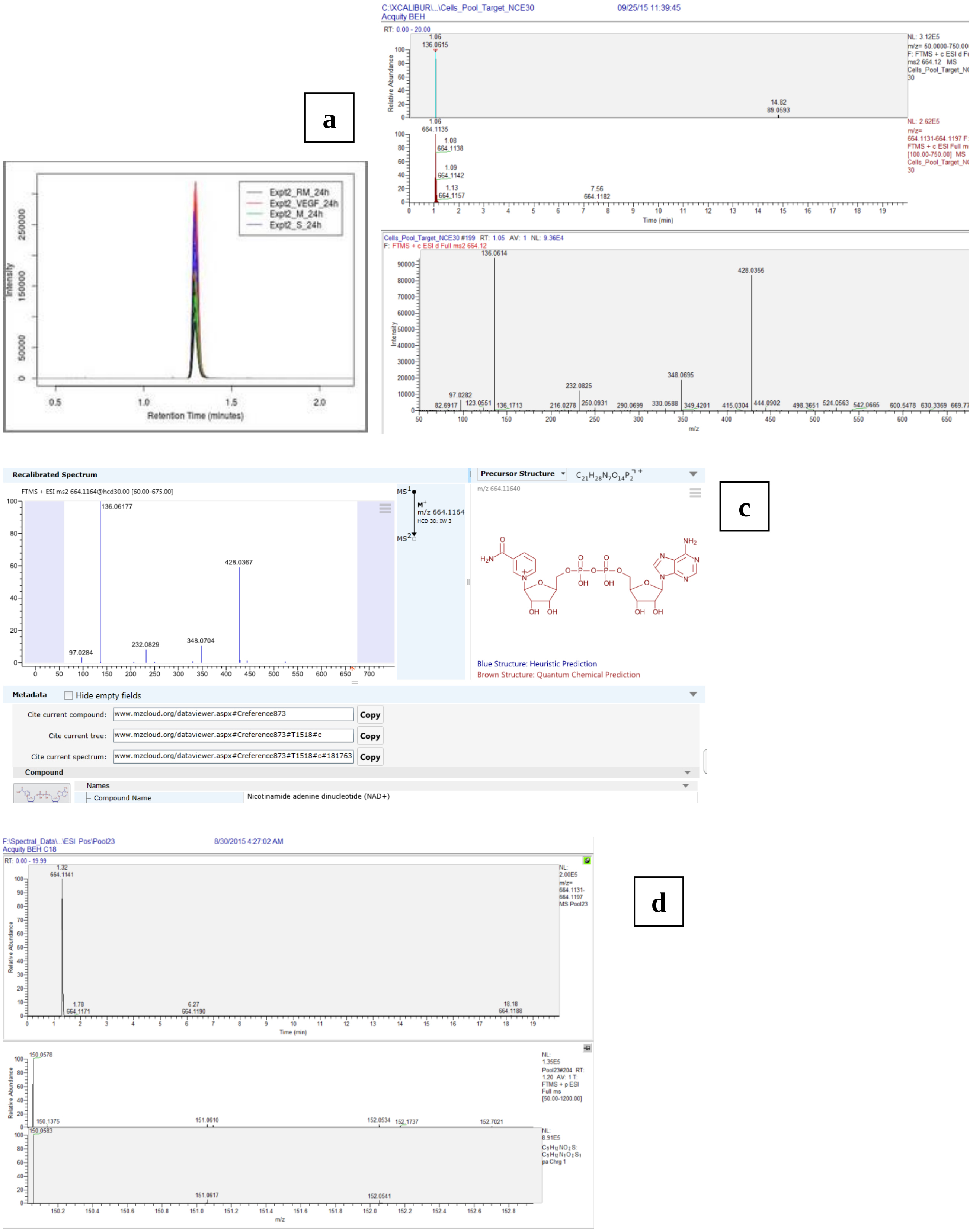

Pantothenic acid (CID match)

a

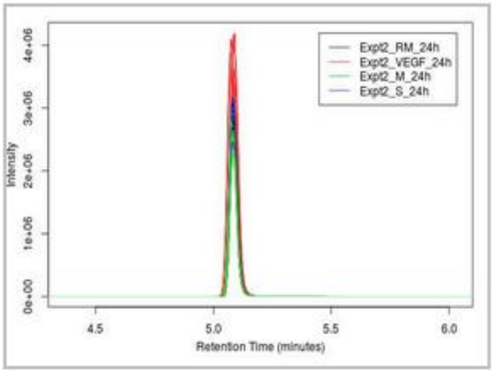

b

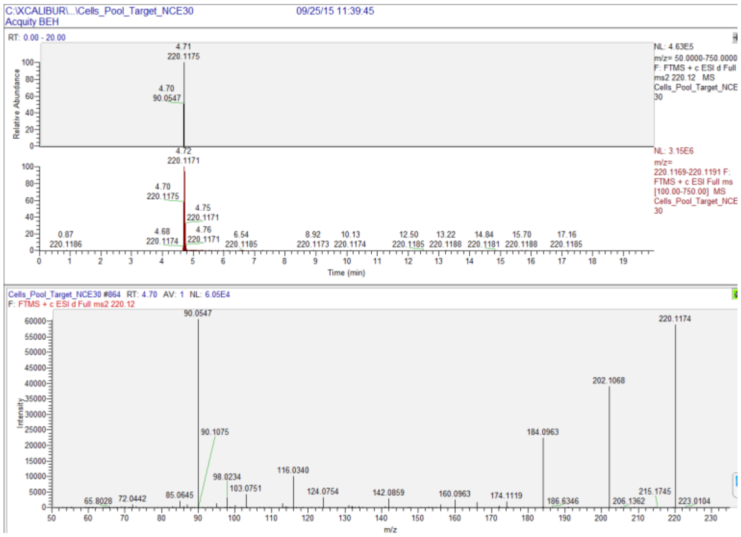

c

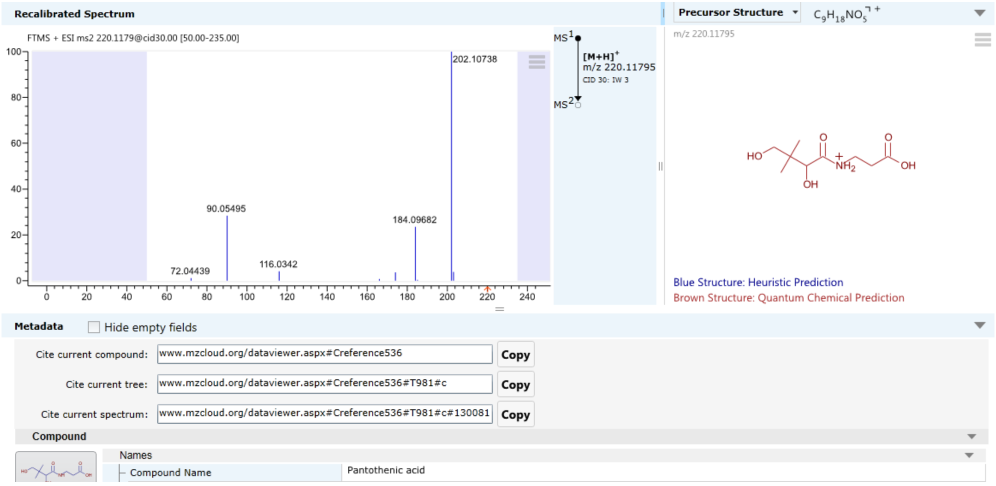

d

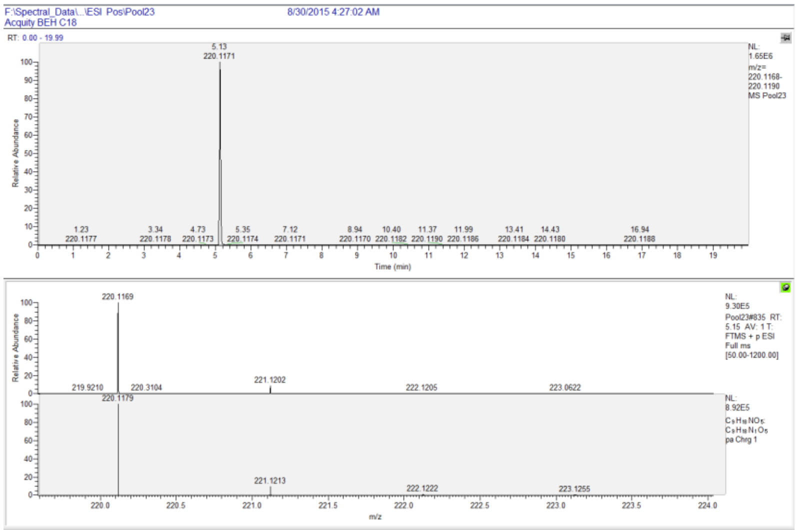

Oleamide

b

a

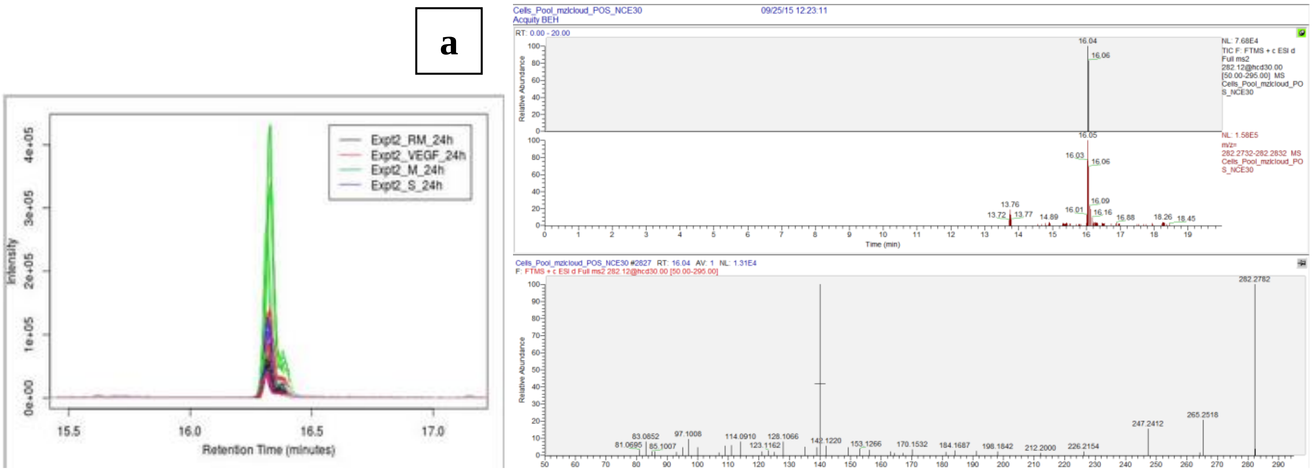
Intensity
Retention Time (minutes)
Expt2_RM_24h
Expt2_VEGF_24h
Expt2_M_24h
Expt2_S_24h
Cells_Pool_mzlcloud_POS_NCE30
09/25/15 12:23:11
Acquity BEH
RT: 0.00 - 20.00
Relative Abundance
Time (min)
NL: 7.68E4
TIC F: FTMS + c ESI d Full ms2 282.12@hcd30.00 [50.00-295.00] MS Cells_Pool_mzlcloud_POS_NCE30
NL: 1.58E5
m/z= 282.2732-282.2832 MS Cells_Pool_mzlcloud_POS_NCE30
Cells_Pool_mzlcloud_POS_NCE30 #2627 RT: 16.04 AV: 1 NL: 1.31E4
F: FTMS + c ESI d Full ms2 282.12@hcd30.00 [50.00-295.00]
282.2782
265.2518
247.2412

c

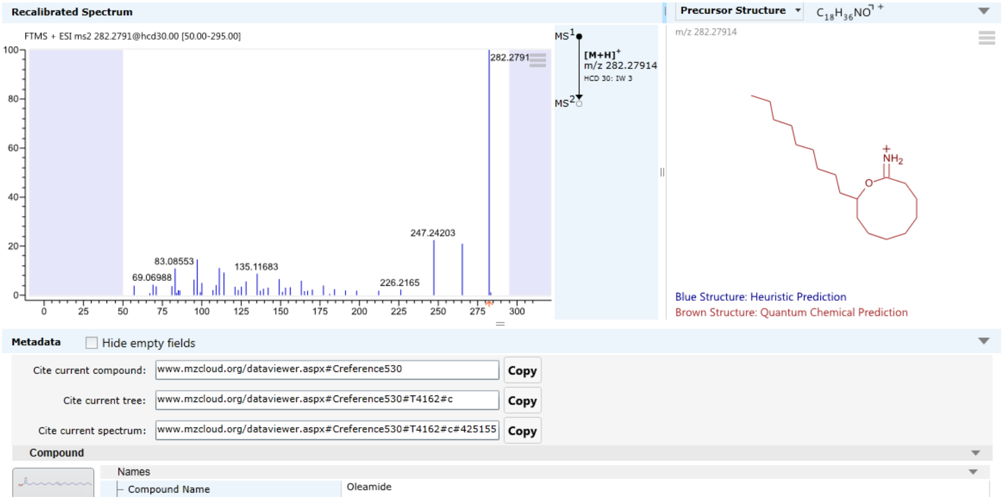
Recalibrated Spectrum
FTMS + ESI ms2 282.2791@hcd30.00 [50.00-295.00]
282.2791
247.24203
226.2165
135.11683
83.08553
69.06988
[M+H]+
m/z 282.27914
HCD 30; IW 3
Precursor Structure
$C_{18}H_{36}NO^{+}$
m/z 282.27914
Blue Structure: Heuristic Prediction
Brown Structure: Quantum Chemical Prediction
Metadata
Hide empty fields
Cite current compound: www.mzcloud.org/dataviewer.aspx#Creference530
Cite current tree: www.mzcloud.org/dataviewer.aspx#Creference530#T4162#c
Cite current spectrum: www.mzcloud.org/dataviewer.aspx#Creference530#T4162#c#425155
Copy
Compound
Names
Compound Name
Oleamide

d

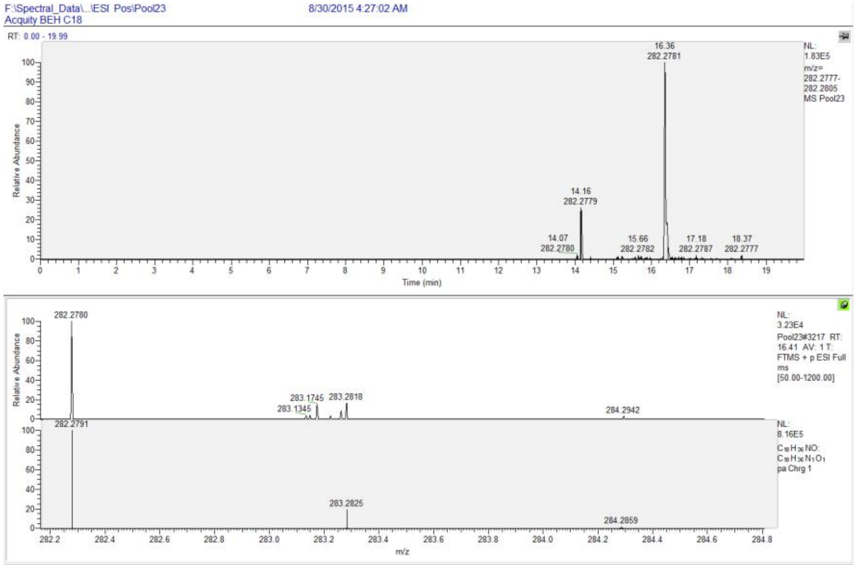
F:\Spectral_Data\...\ESI Pos\Pool23
8/30/2015 4:27:02 AM
Acquity BEH C18
RT: 0.00 - 19.99
16.36
282.2781
14.16
282.2779
Relative Abundance
Time (min)
NL: 1.83E5
m/z= 282.2777-282.2805 MS Pool23
282.2780
283.1745
283.2818
284.2942
NL: 3.23E4
Pool23#3217 RT: 16.41 AV: 1 T: FTMS + p ESI Full ms [50.00-1200.00]
282.2791
283.2825
284.2859
NL: 8.16E5
m/z

Ƴ-L-Glutamyl-L-Glutamic acid

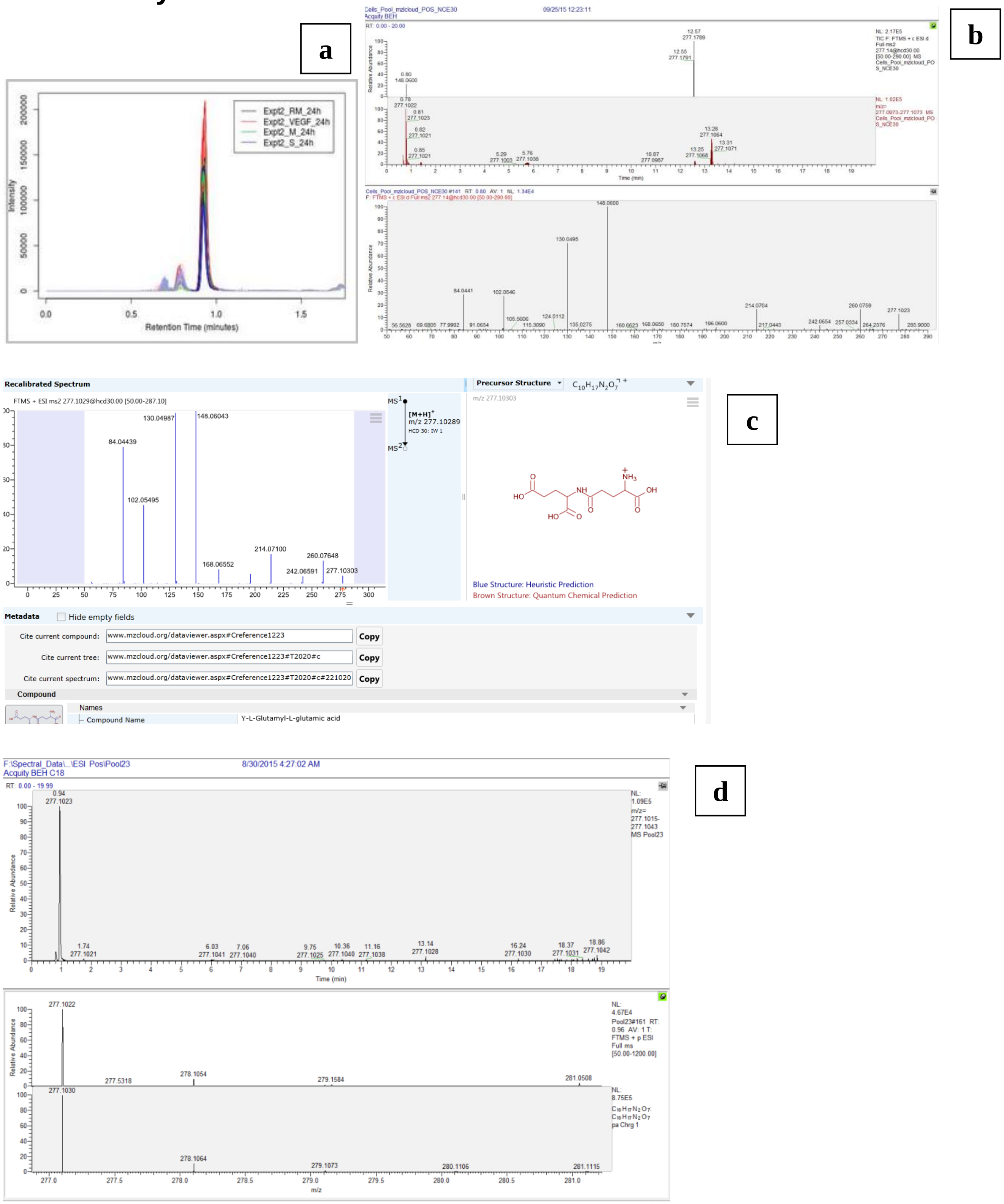

Inosine

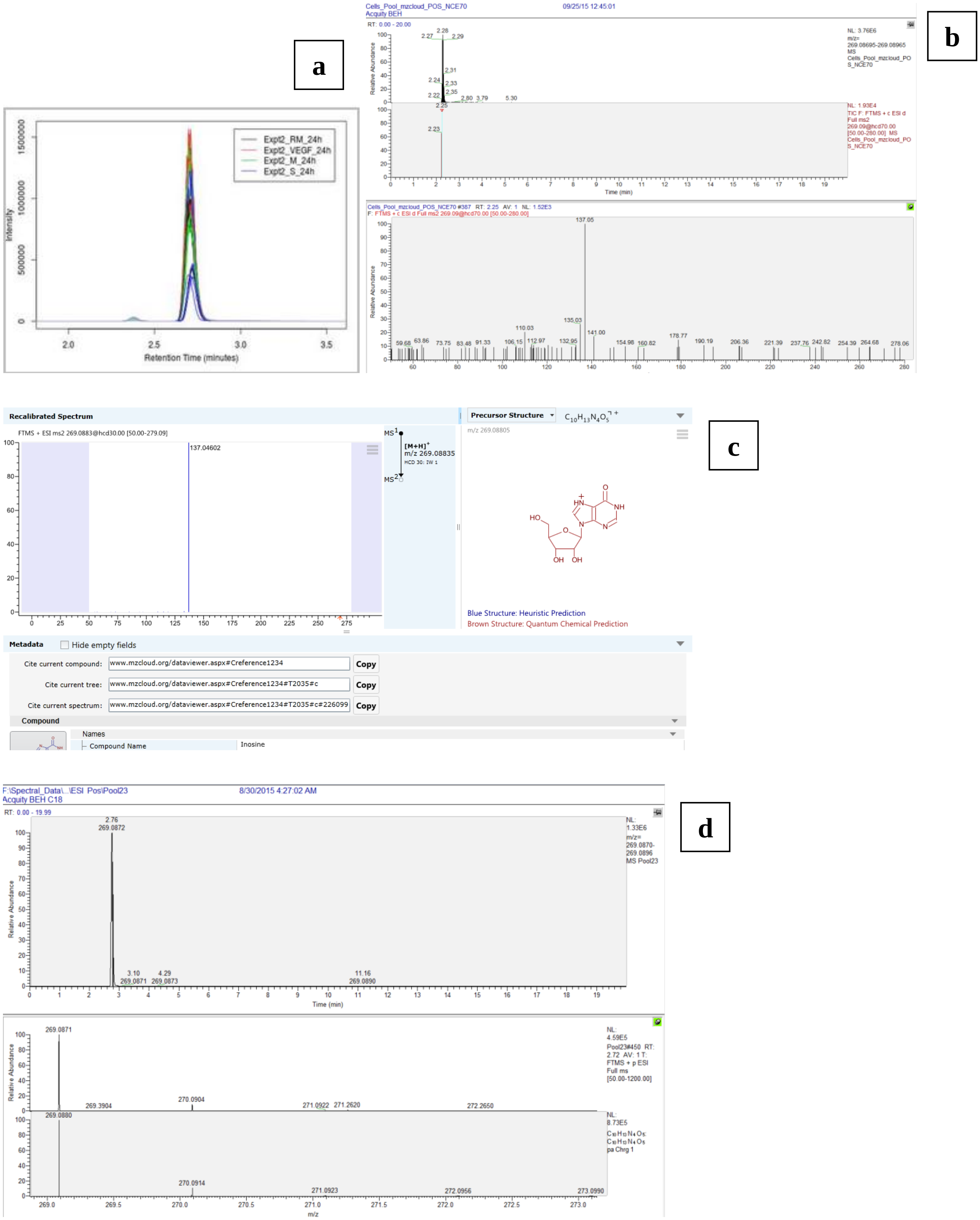

a
b
c
d
Expt2_RM_24h
Expt2_VEGF_24h
Expt2_M_24h
Expt2_S_24h
Intensity
Retention Time (minutes)
Cells_Pool_mzcloud_POS_NCE70
Acquity BEH
09/25/15 12:45:01
Relative Abundance
Time (min)
Cells_Pool_mzcloud_POS_NCE70 #387 RT: 2.25 AV: 1 NL: 1.52E3
F: FTMS + c ESI d Full ms2 269.09@hcd70.00 [50.00-280.00]
137.05
Recalibrated Spectrum
FTMS + ESI ms2 269.0883@hcd30.00 [50.00-279.09]
137.04602
[M+H]+
m/z 269.08835
HCD 30: IW 1
Precursor Structure
m/z 269.08805
Blue Structure: Heuristic Prediction
Brown Structure: Quantum Chemical Prediction
Metadata
Hide empty fields
Cite current compound:
www.mzcloud.org/dataviewer.aspx#Creference1234
Cite current tree:
www.mzcloud.org/dataviewer.aspx#Creference1234#T2035#c
Cite current spectrum:
www.mzcloud.org/dataviewer.aspx#Creference1234#T2035#c#226099
Copy
Compound
Names
Compound Name
Inosine
F:\Spectral_Data\...\ESI Pos\Pool23
Acquity BEH C18
8/30/2015 4:27:02 AM
2.76
269.0872
Time (min)
269.0871
269.0880
m/z

Spermine

a. Peak not shown in XCMS Online as $p > 0.05$.

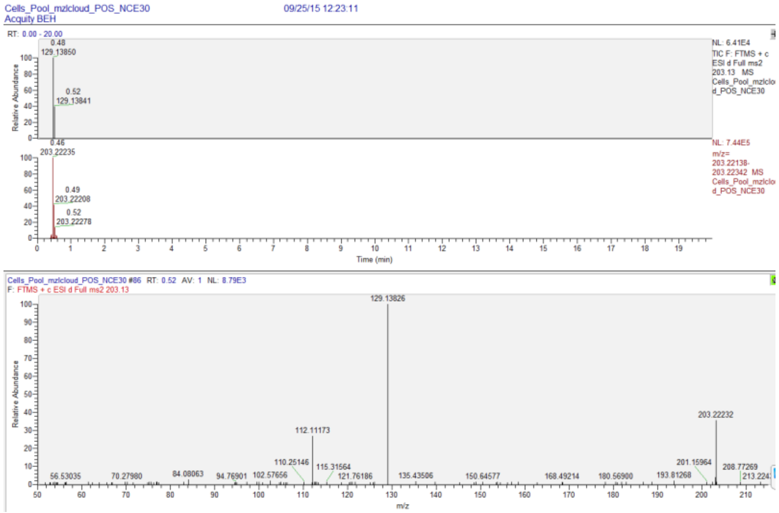

b

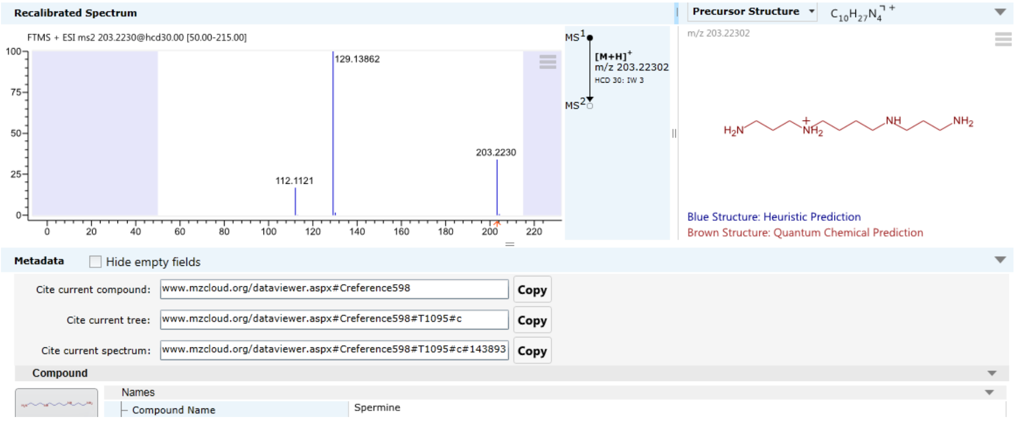

c

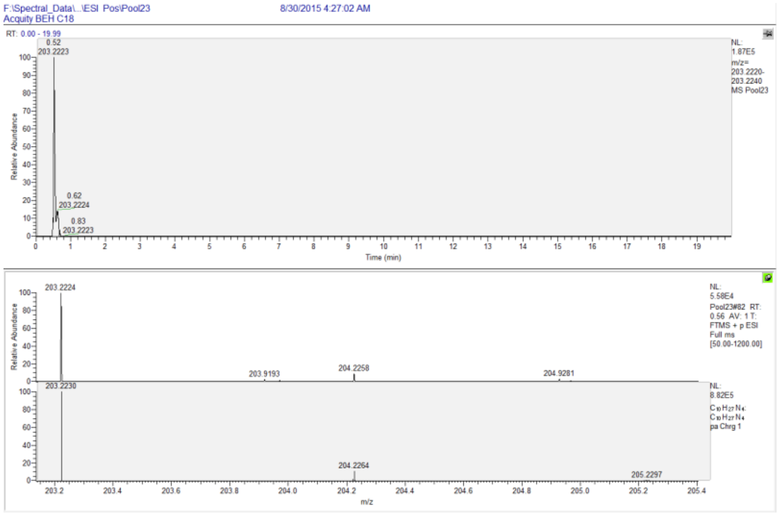

d

Indole 3-acrylic acid / *trans* 3-Indole acrylic acid

a

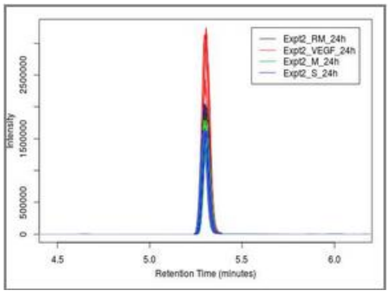

b

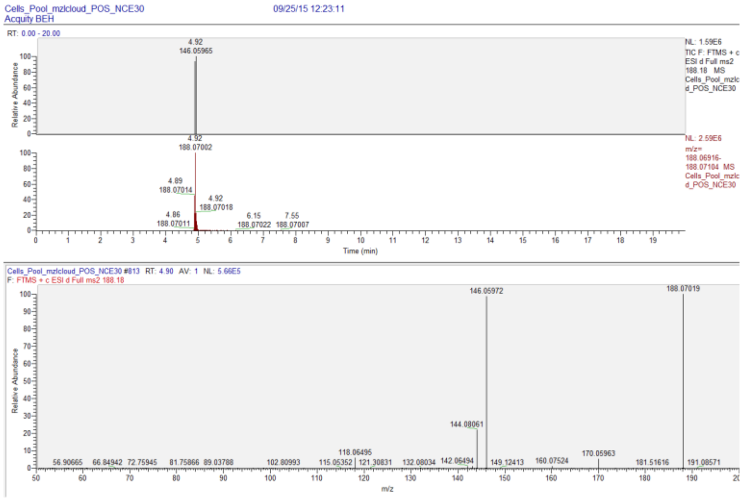

c

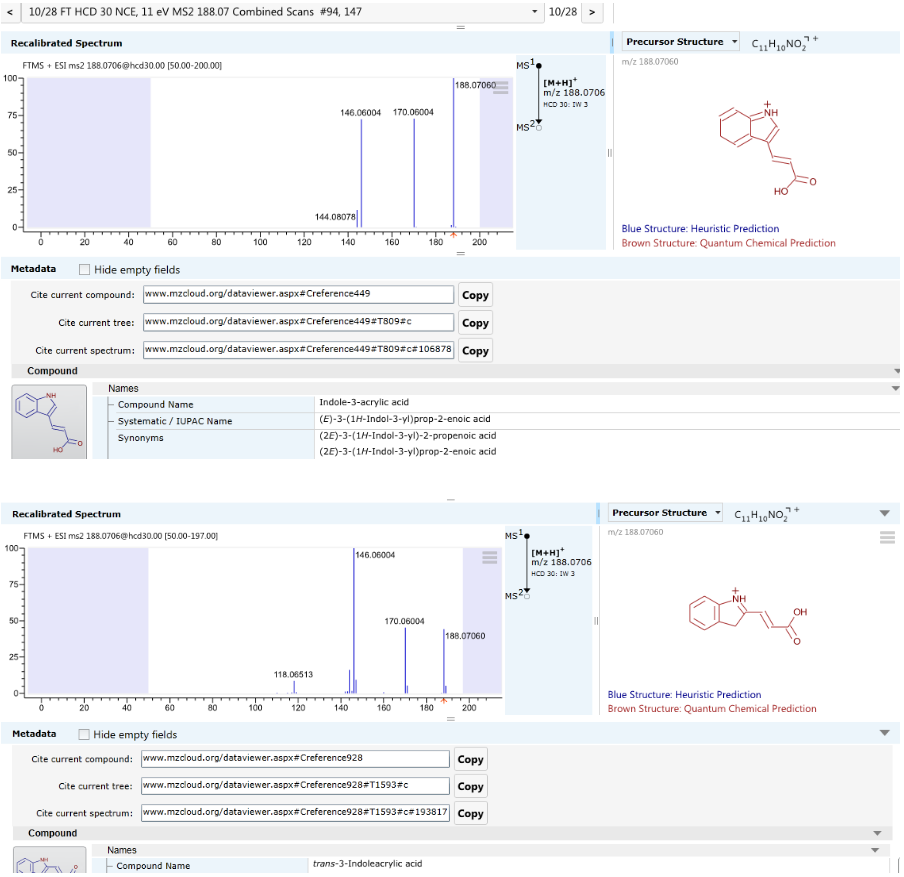

d

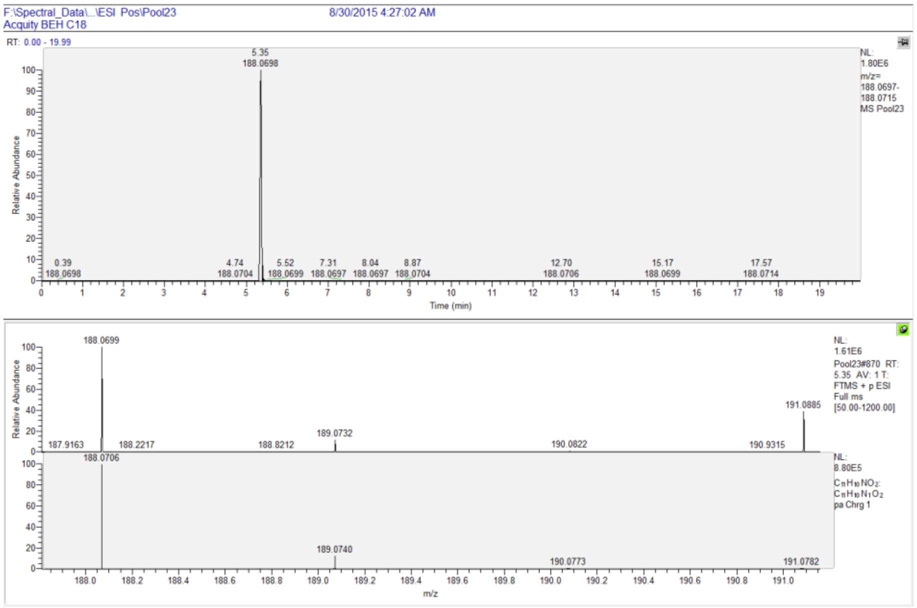
F:\Spectral_Data\...\ESI Pos\Pool23
8/30/2015 4:27:02 AM
Acquity BEH C18
RT: 0.00 - 19.99
5.35
188.0698
NL:
1.80E6
m/z=
188.0697-
188.0715
MS Pool23
Relative Abundance
0.39 188.0698
4.74 188.0704
5.52 188.0699
7.31 188.0697
8.04 188.0697
8.87 188.0704
12.70 188.0706
15.17 188.0699
17.57 188.0714
Time (min)
188.0699
NL:
1.61E6
Pool23#870 RT: 5.35 AV: 1 T: FTMS + p ESI Full ms [50.00-1200.00]
187.9163
188.2217
188.8212
189.0732
190.0822
190.9315
191.0885
188.0706
NL:
8.80E5
$C_{11}H_{10}NO_2$:
$C_{11}H_{10}N_1O_2$
pa Chrg 1
189.0740
190.0773
191.0782
m/z

Cysteinylglycine

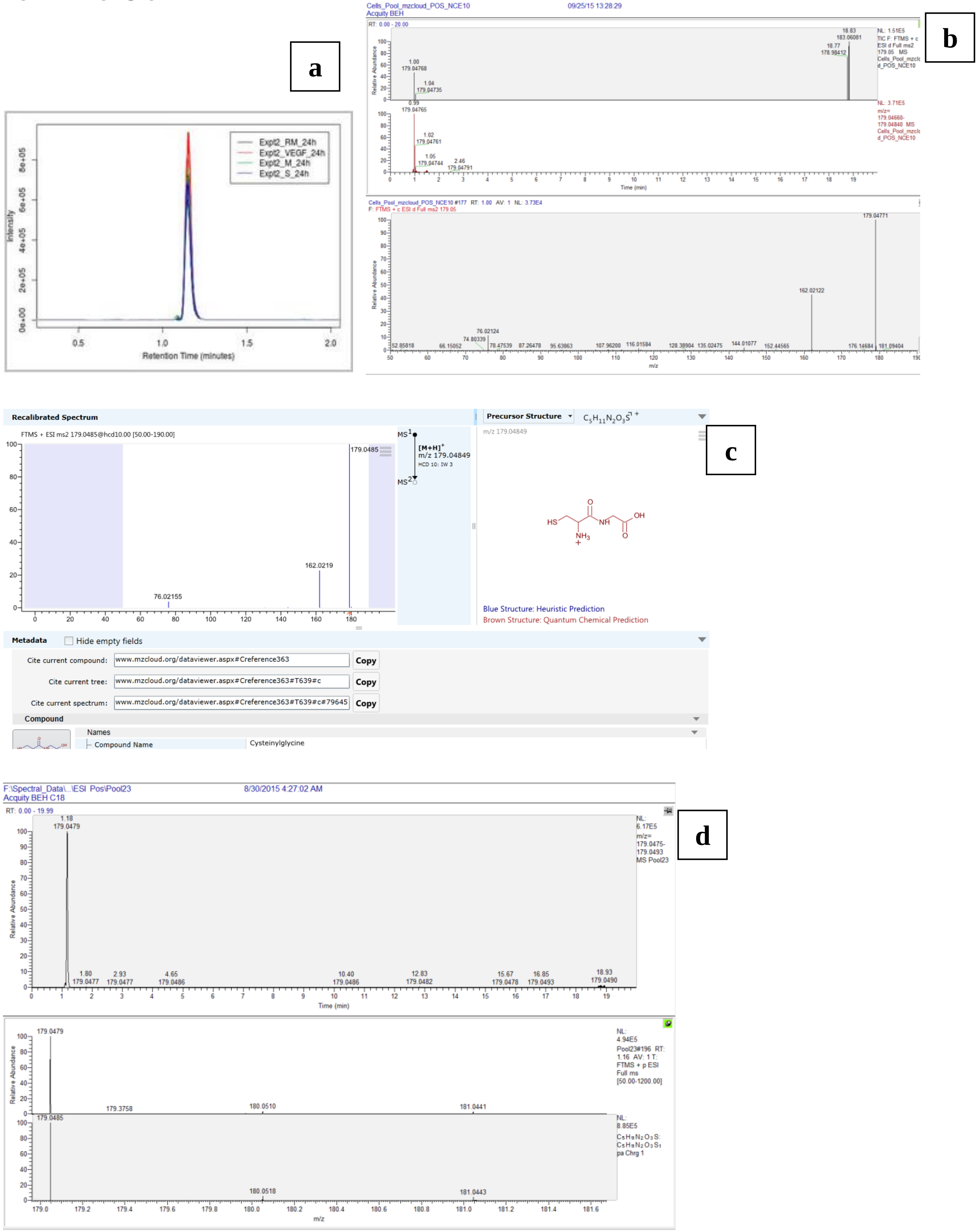

Guanine

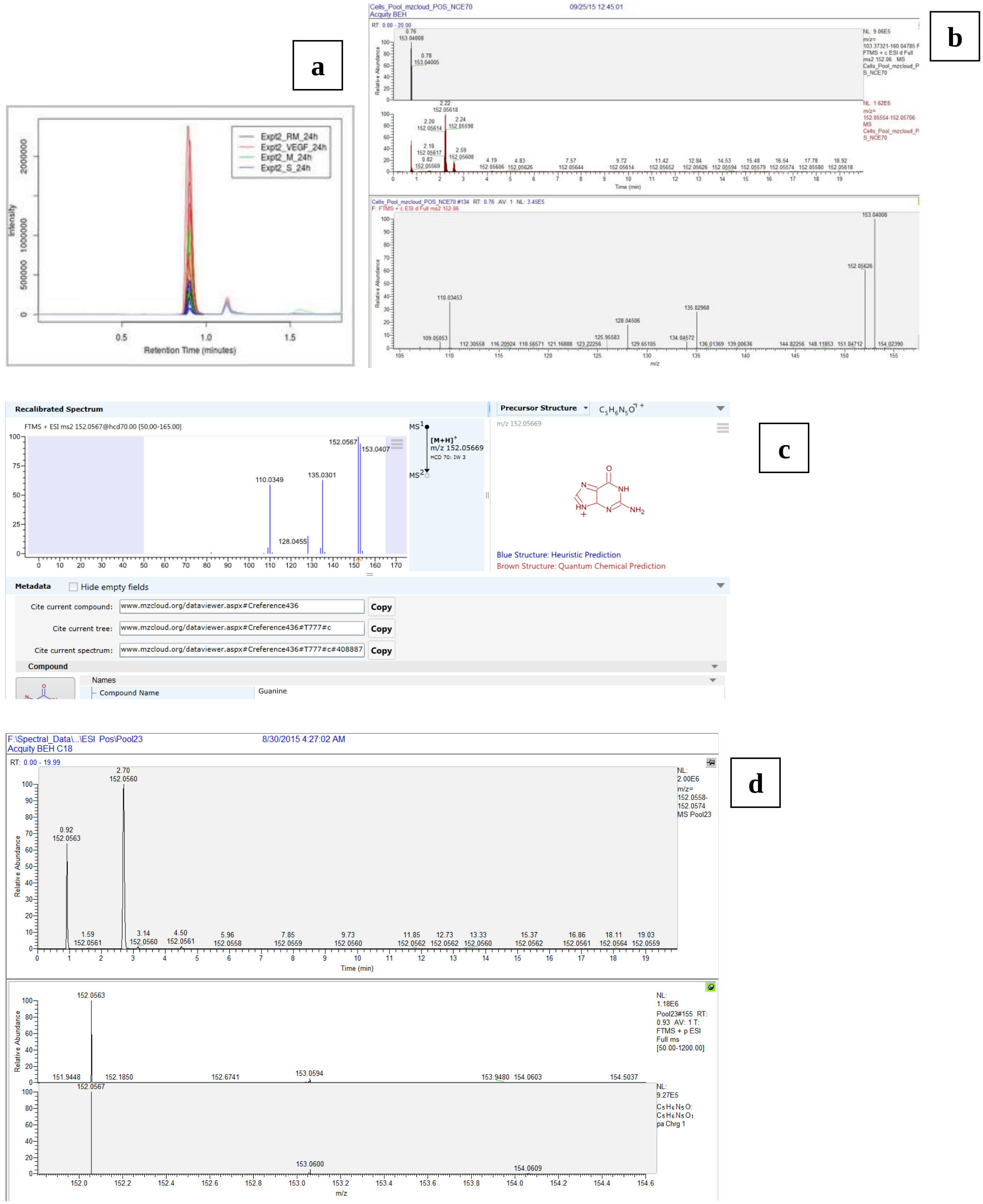

a
Expt2_RM_24h
Expt2_VEGF_24h
Expt2_M_24h
Expt2_S_24h
Intensity
Retention Time (minutes)
b
Cells_Pool_mzcloud_POS_NCE70
Acquity BEH
09/25/15 12:45:01
Relative Abundance
Time (min)
m/z
c
Recalibrated Spectrum
FTMS + ESI ms2 152.0567@hcd70.00 [50.00-165.00]
152.0567
153.0407
135.0301
110.0349
128.0455
[M+H]+
m/z 152.05669
Precursor Structure
m/z 152.05669
Blue Structure: Heuristic Prediction
Brown Structure: Quantum Chemical Prediction
Metadata
Hide empty fields
Cite current compound: www.mzcloud.org/dataviewer.aspx#Creference436
Cite current tree: www.mzcloud.org/dataviewer.aspx#Creference436#T777#c
Cite current spectrum: www.mzcloud.org/dataviewer.aspx#Creference436#T777#c#408887
Copy
Compound
Names
Compound Name
Guanine
d
F:\Spectral_Data\...\ESI Pos\Pool23
Acquity BEH C18
8/30/2015 4:27:02 AM
RT: 0.00 - 19.99
NL: 2.00E6 m/z= 152.0558-152.0574 MS Pool23
2.70 152.0560
0.92 152.0563
Relative Abundance
Time (min)
NL: 1.18E6 Pool23#155 RT: 0.93 AV: 1 T: FTMS + p ESI Full ms [50.00-1200.00]
152.0563
152.0567
153.0594
153.0600
154.0609
NL: 9.27E5
m/z

Creatine

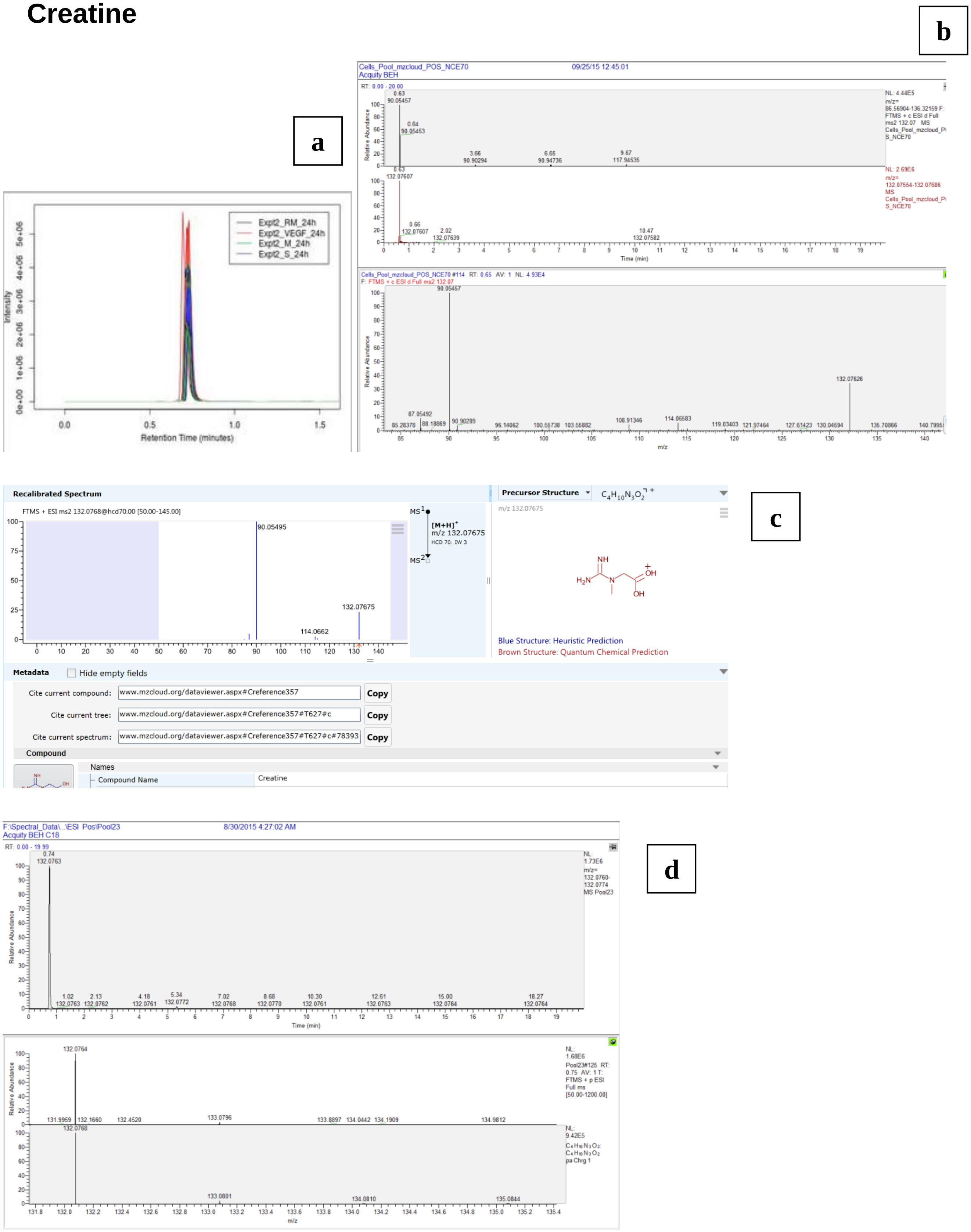

Hypoxanthine (RT 1.19 min)

b

a

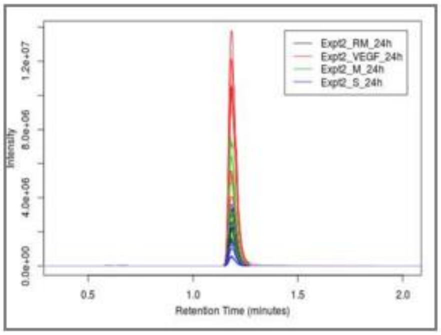

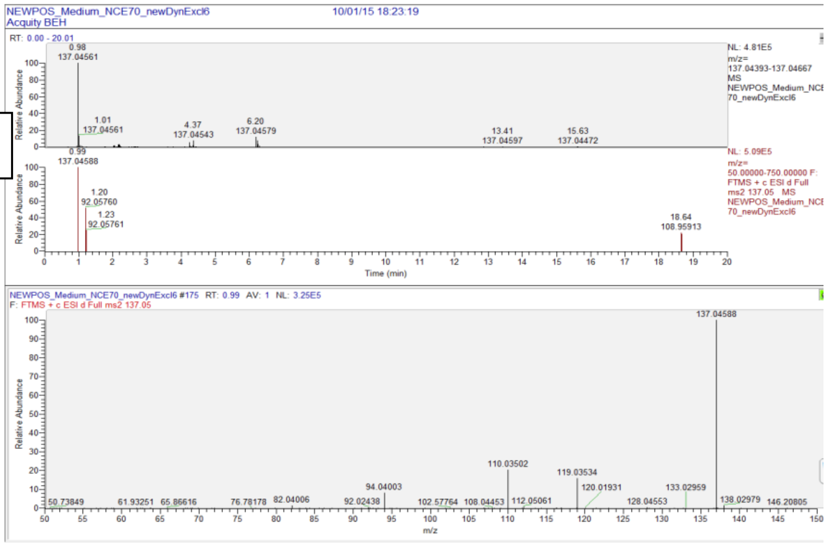

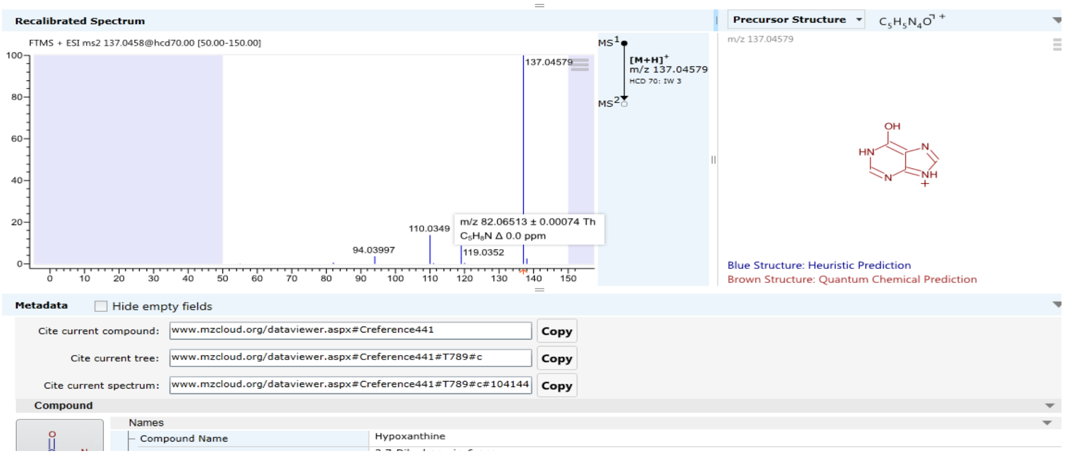

c

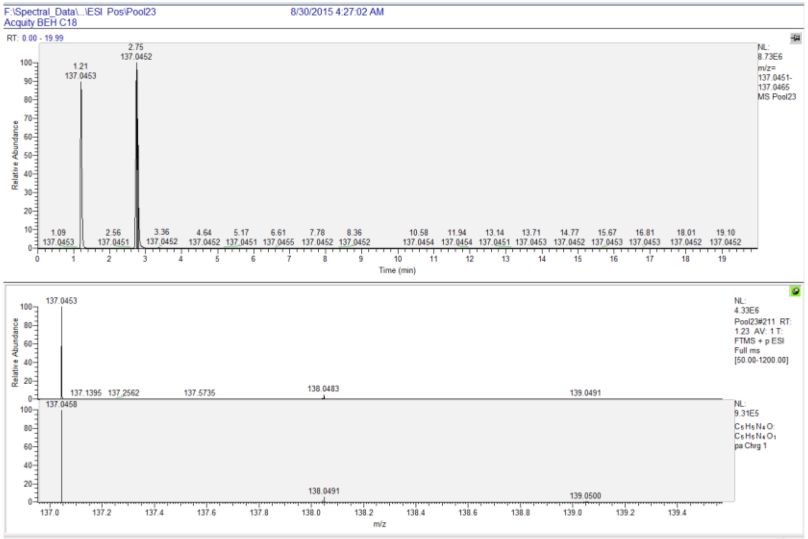

d

Acetyl L-Carnitine

b

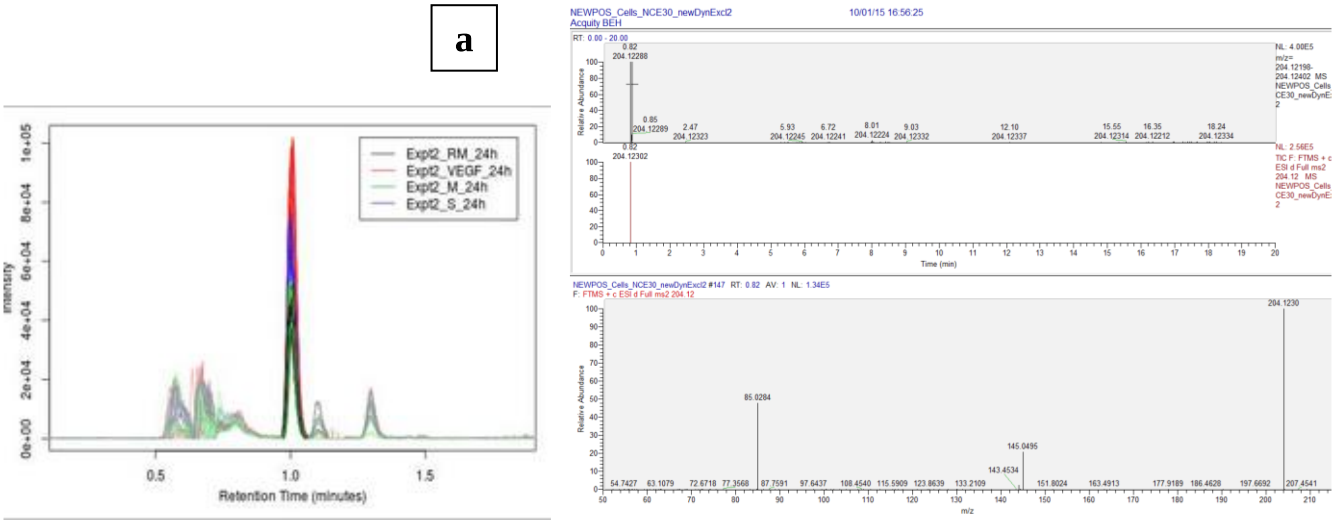
a
Expt2_RM_24h
Expt2_VEGF_24h
Expt2_M_24h
Expt2_S_24h
Intensity
Retention Time (minutes)
NEWPOS_Cells_NCE30_newDynExcl2
Acquity BEH
10/01/15 16:56:25
Relative Abundance
Time (min)
m/z

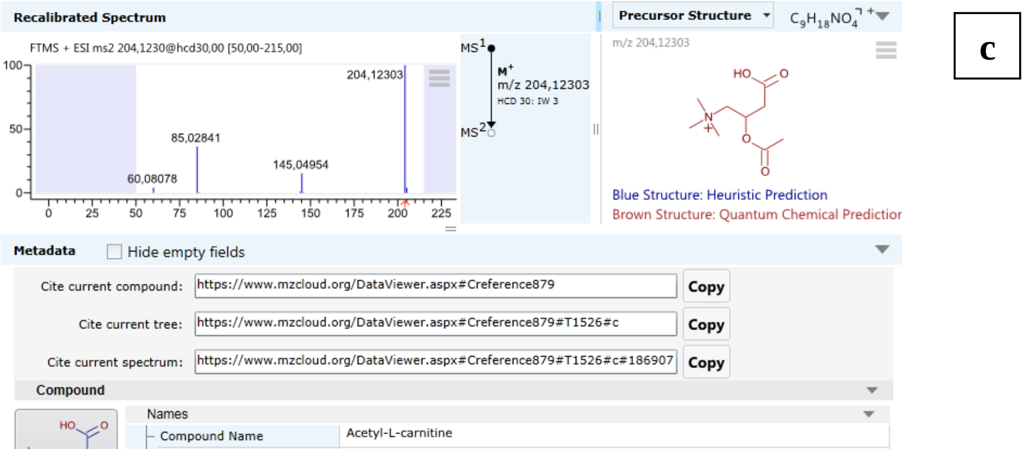
c
Recalibrated Spectrum
FTMS + ESI ms2 204,1230@hcd30,00 [50,00-215,00]
204,12303
85,02841
145,04954
60,08078
MS1
M+
m/z 204,12303
HCD 30: IW 3
MS2
Precursor Structure
m/z 204,12303
Blue Structure: Heuristic Prediction
Brown Structure: Quantum Chemical Predictior
Metadata
Hide empty fields
Cite current compound: https://www.mzcloud.org/DataViewer.aspx#Creference879
Cite current tree: https://www.mzcloud.org/DataViewer.aspx#Creference879#T1526#c
Cite current spectrum: https://www.mzcloud.org/DataViewer.aspx#Creference879#T1526#c#186907
Copy
Compound
Names
Compound Name
Acetyl-L-carnitine

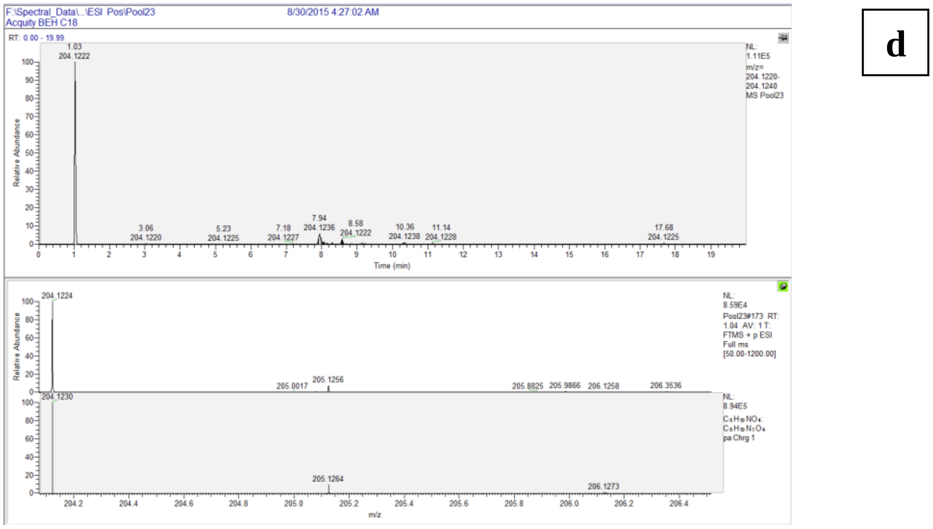
d
F:\Spectral_Data\...\ESI Pos\Pool23
Acquity BEH C18
8/30/2015 4:27:02 AM
Relative Abundance
Time (min)
m/z

L-Aspartate

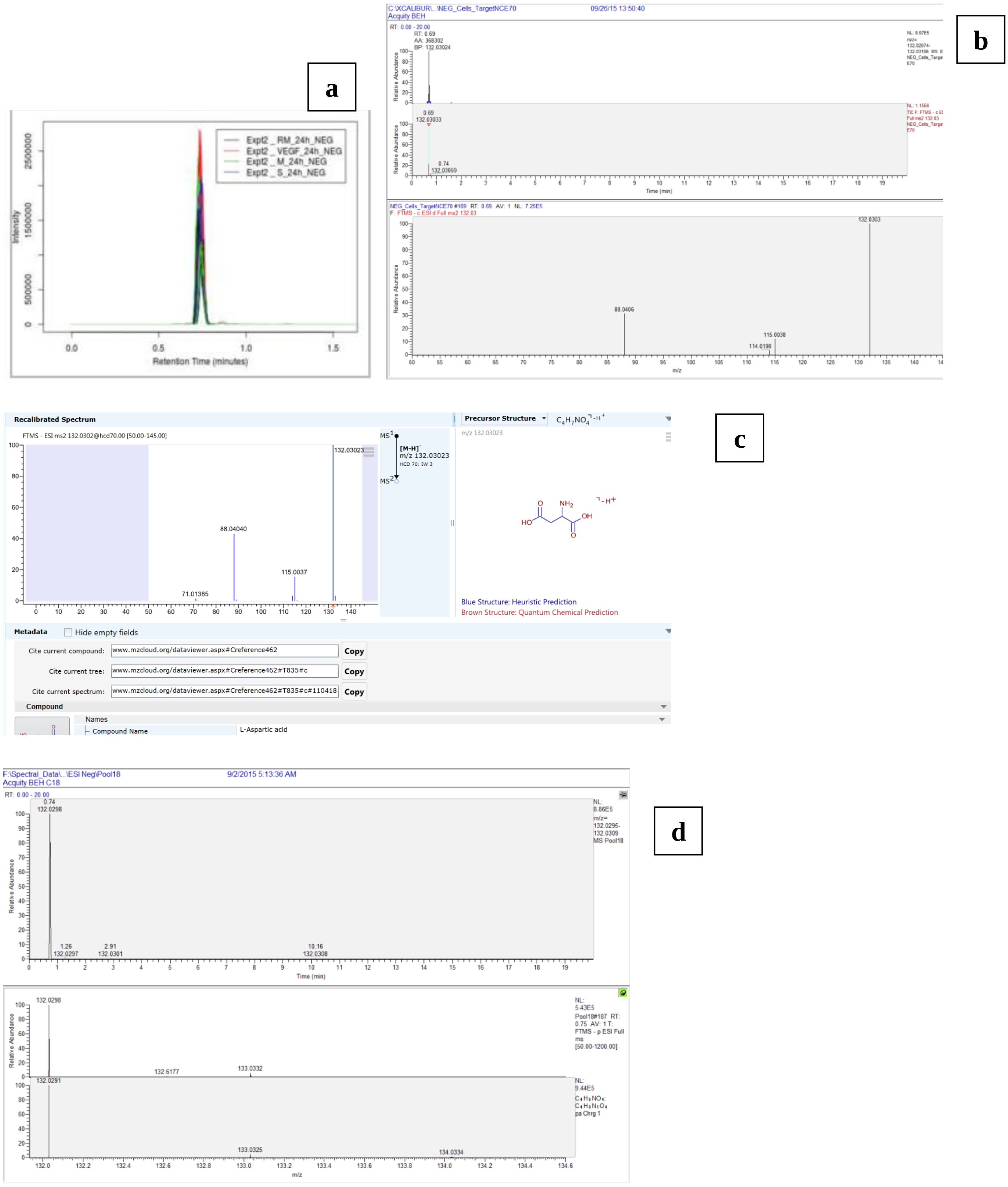

L- Glutamate (ESI-)

a. Peak not displayed in XCMS Online as p > 0.05.

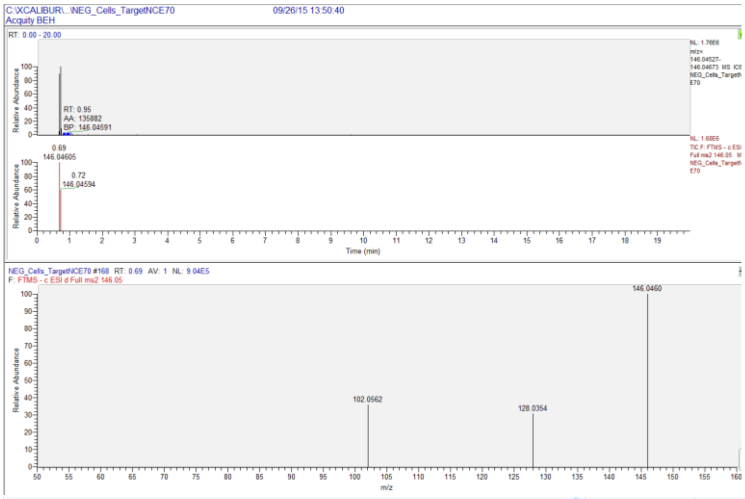

b

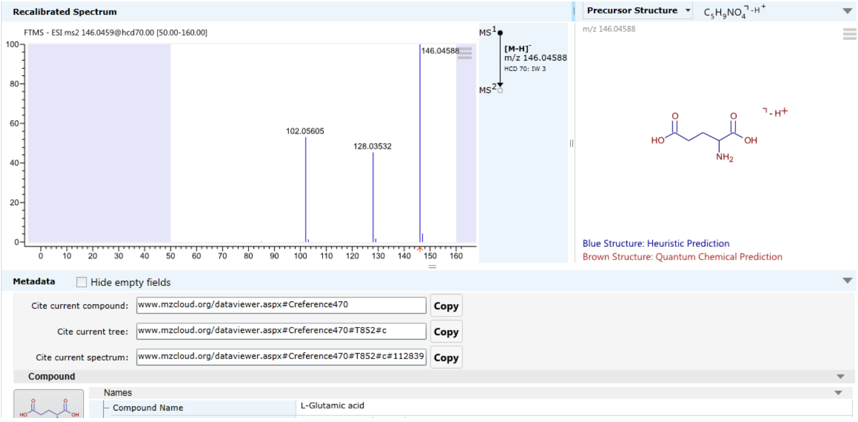

c

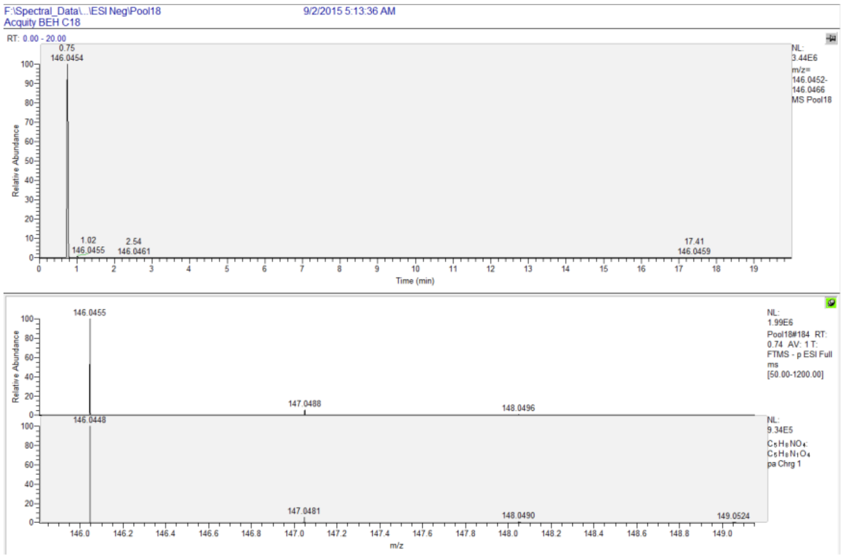

d

Glycerol-3-phosphate

a. Peak not displayed in XCMS Online as $p > 0.05$.

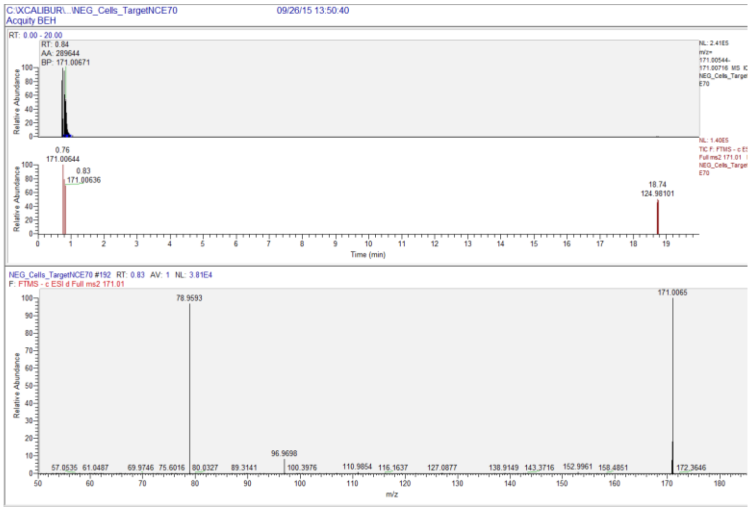

b

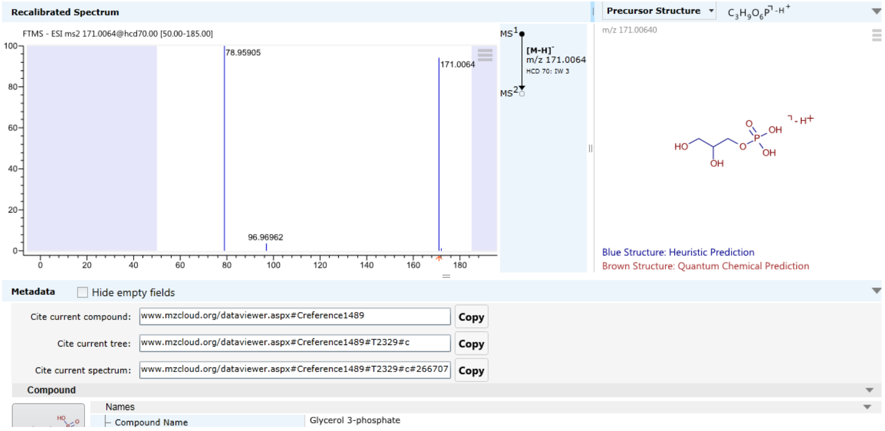

c

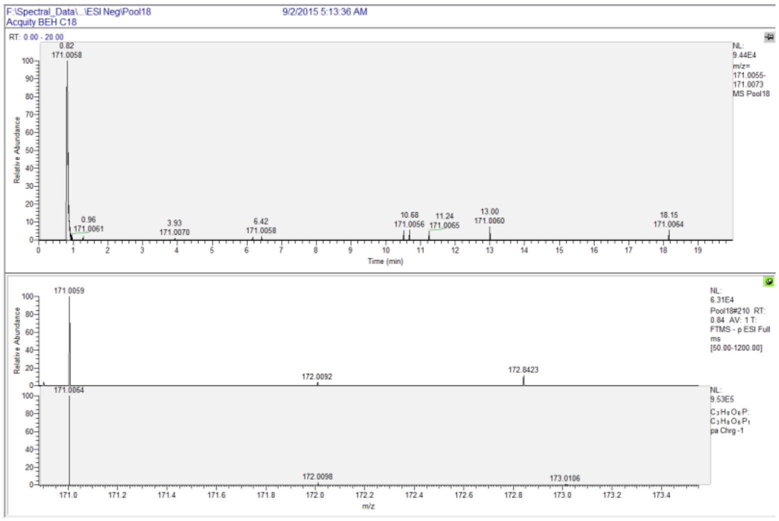

d

Pantothenic acid (ESI-)

a

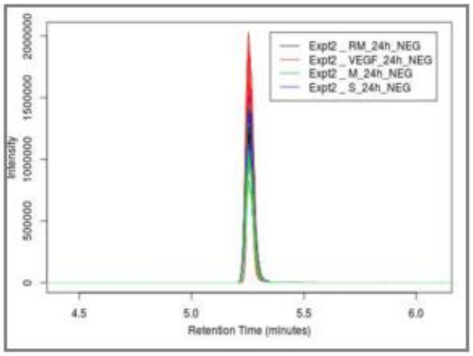

b

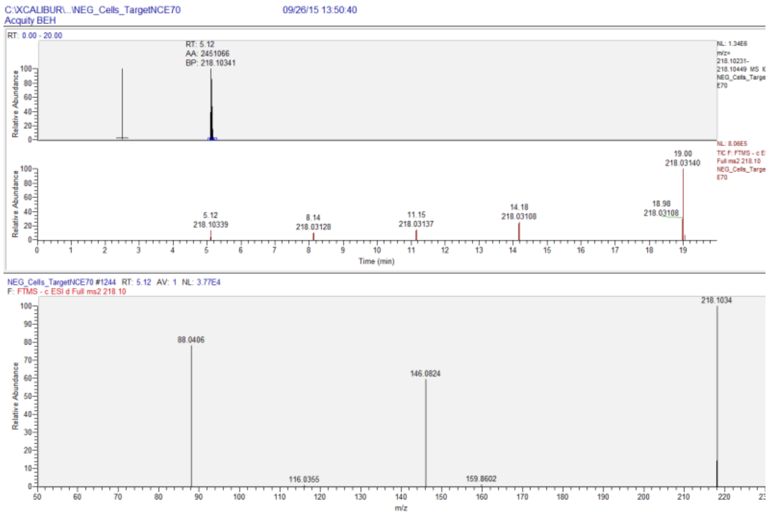

c

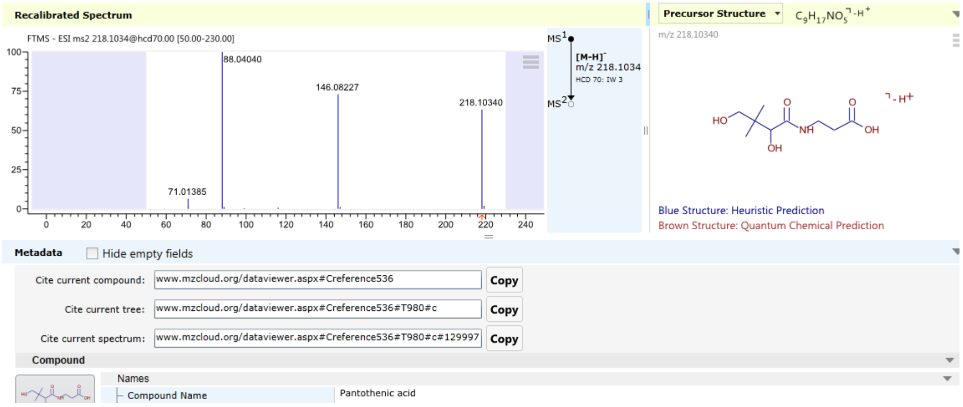

d

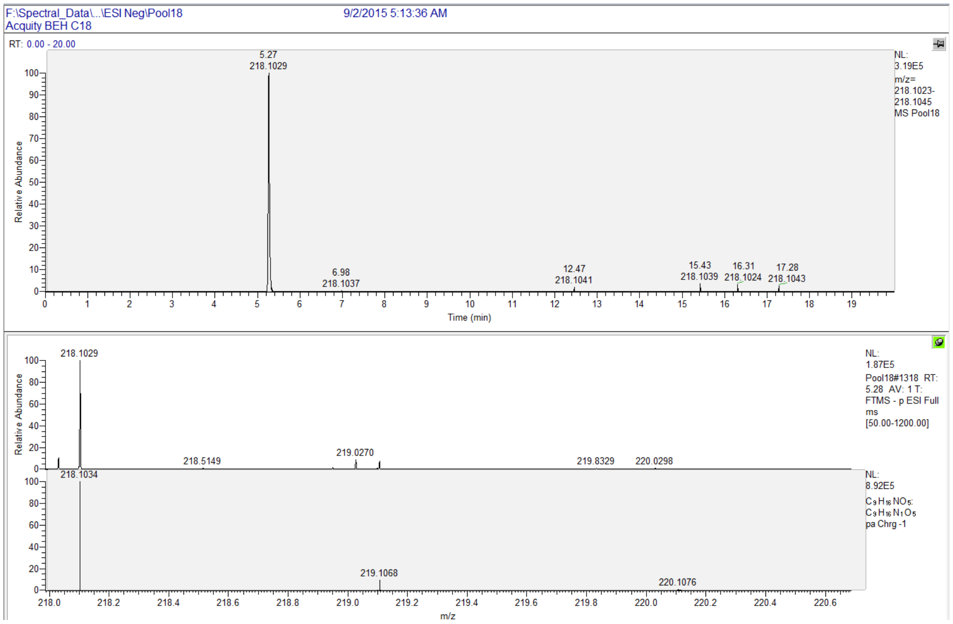

Uridine

a

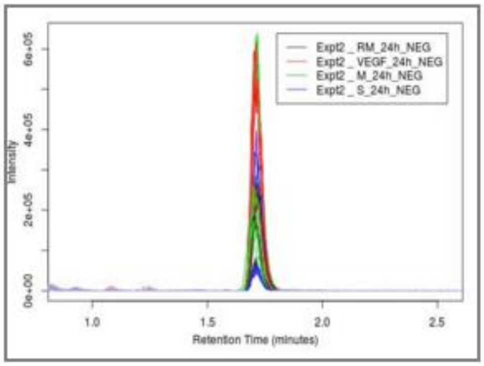

b

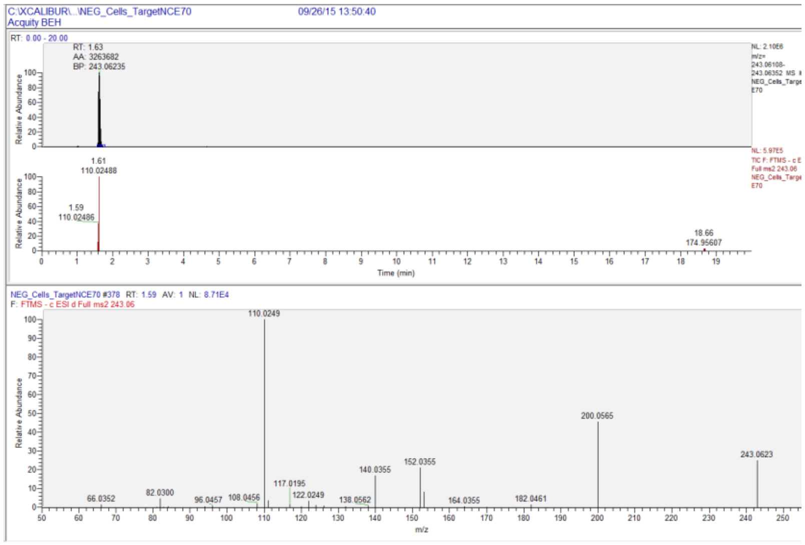

c

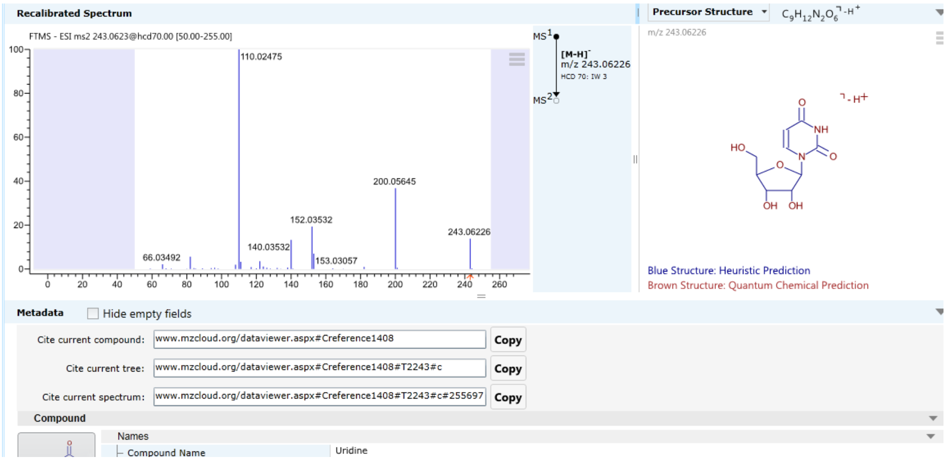

d

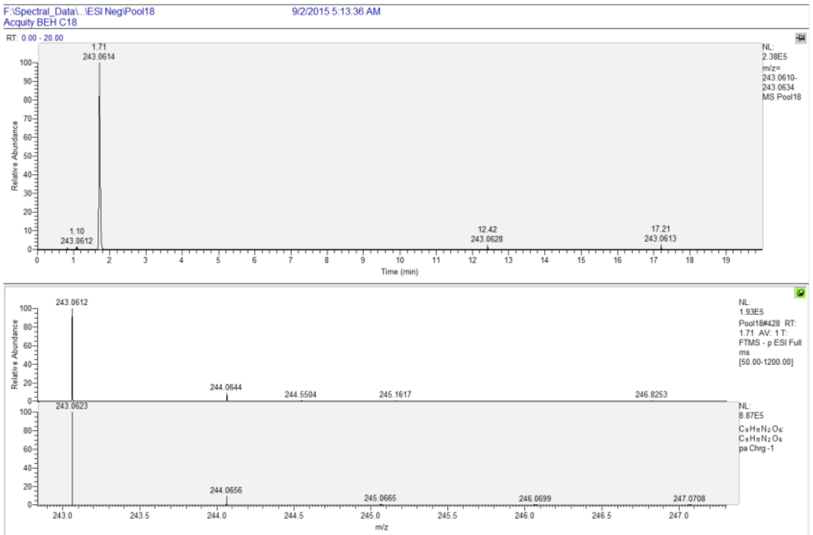

L-Glutathione reduced (ESI-)

b

a

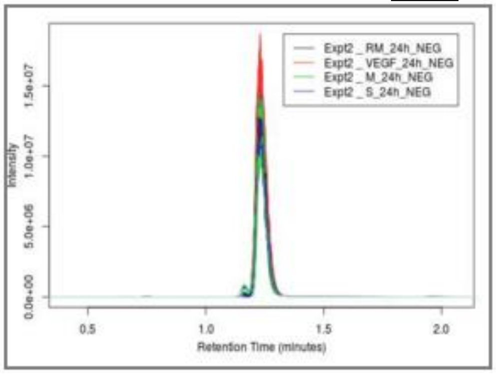

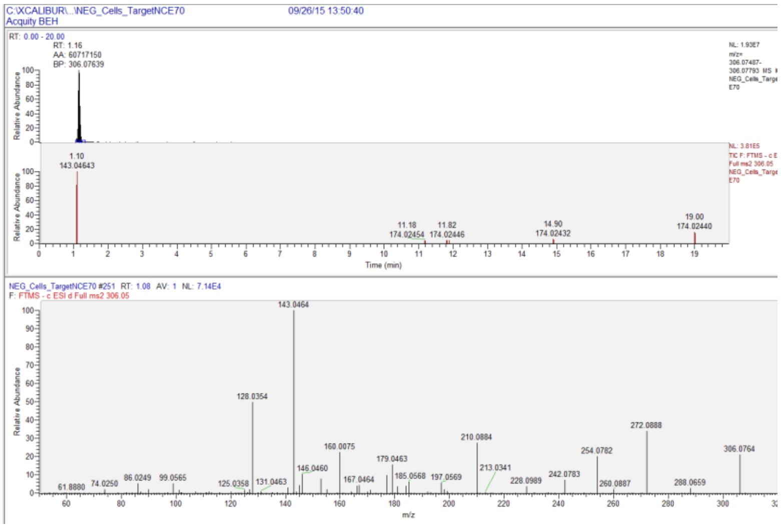

c

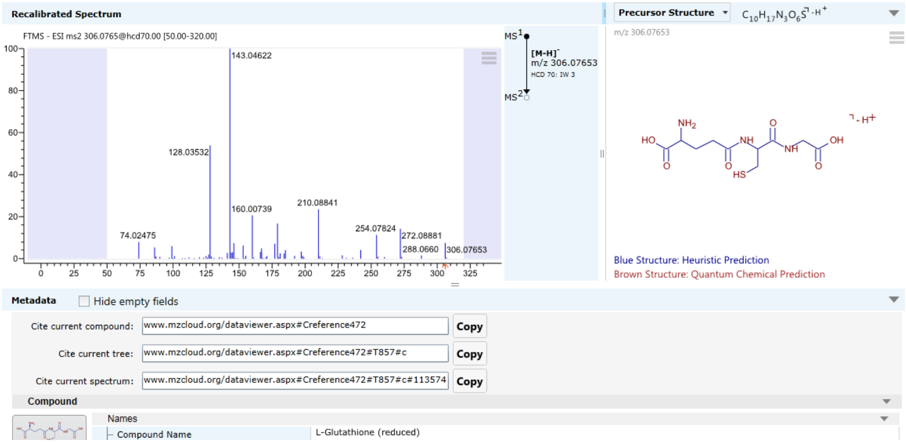

d

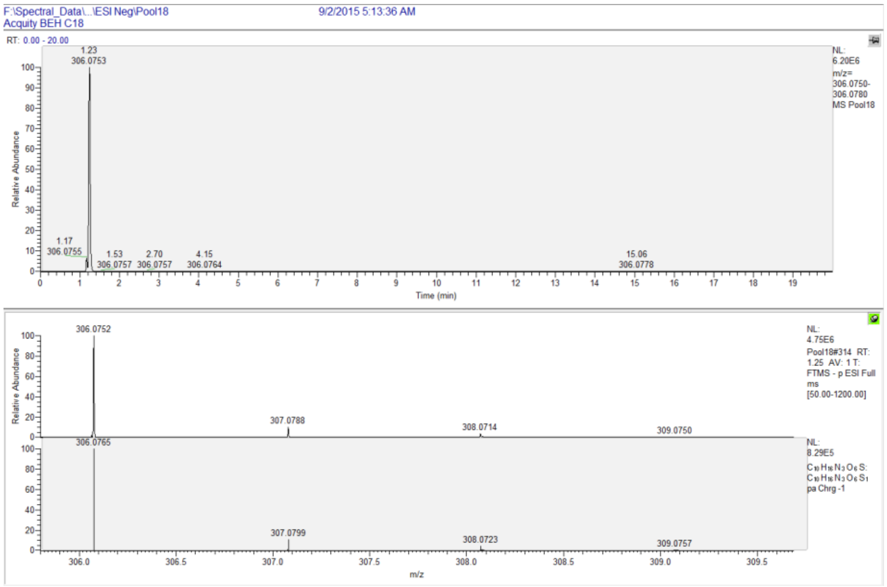

Uridine monophosphate (UMP)

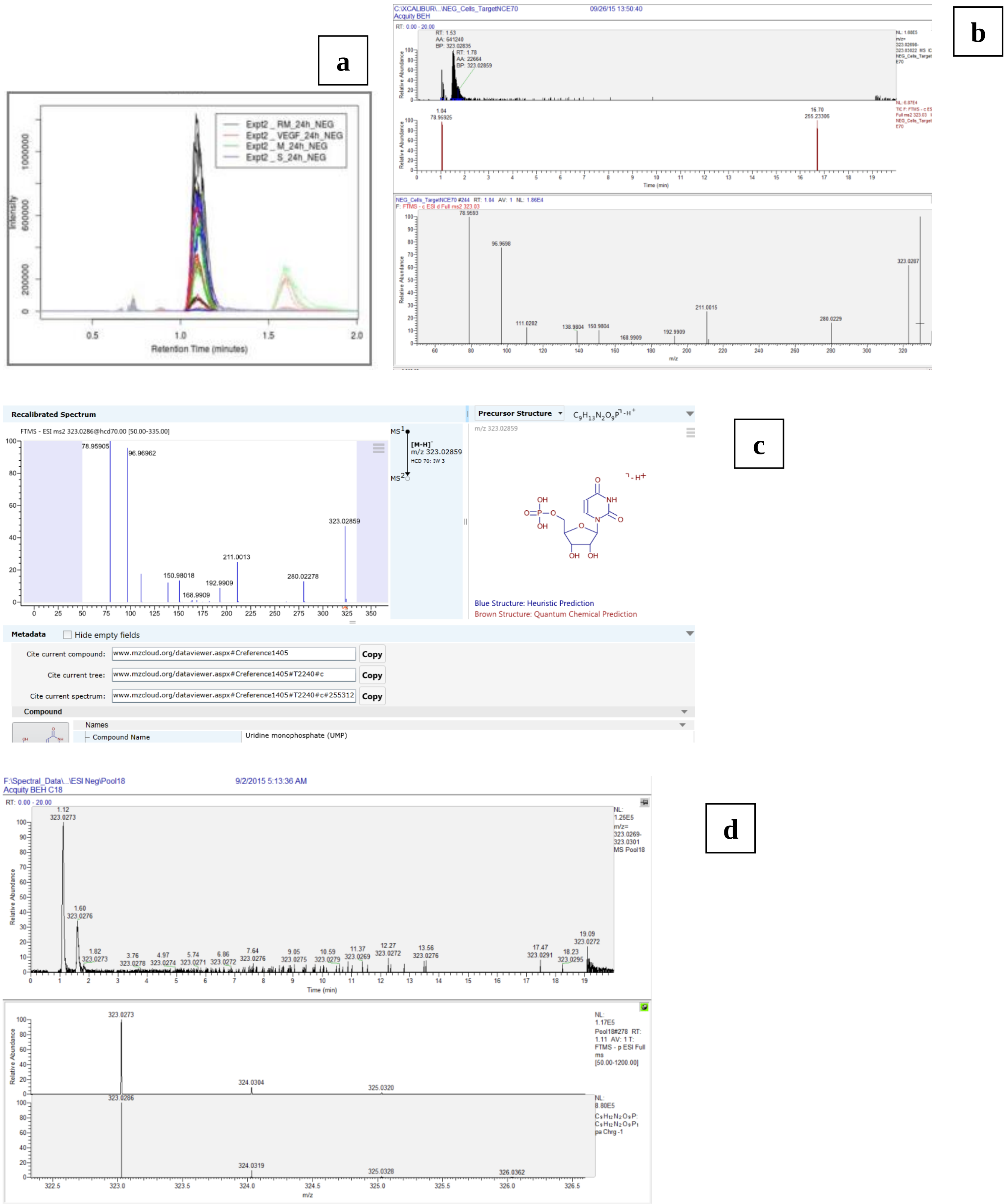

a
b
c
d
Expt2 _ RM_24h_NEG
Expt2 _ VEGF_24h_NEG
Expt2 _ M_24h_NEG
Expt2 _ S_24h_NEG
Intensity
Retention Time (minutes)
C:\XCALIBUR\..\NEG_Cells_TargetNCE70
09/26/15 13:50:40
Acquity BEH
RT: 1.63
AA: 641240
BP: 323.02835
RT: 1.78
AA: 22664
BP: 323.02859
1.04
78.95925
16.70
255.23306
Relative Abundance
Time (min)
NEG_Cells_TargetNCE70 #244 RT: 1.04 AV: 1 NL: 1.86E4
78.9593
96.9698
111.0202
138.9804
150.9804
168.9909
192.9909
211.0015
280.0229
323.0287
m/z
Recalibrated Spectrum
FTMS - ESI ms2 323.0286@hcd70.00 [50.00-335.00]
78.95905
96.96962
150.98018
168.9909
192.9909
211.0013
280.02278
323.02859
[M-H]⁻
m/z 323.02859
Precursor Structure
C9H13N2O9P -H+
m/z 323.02859
Blue Structure: Heuristic Prediction
Brown Structure: Quantum Chemical Prediction
Metadata
Hide empty fields
Cite current compound:
www.mzcloud.org/dataviewer.aspx#Creference1405
Cite current tree:
www.mzcloud.org/dataviewer.aspx#Creference1405#T2240#c
Cite current spectrum:
www.mzcloud.org/dataviewer.aspx#Creference1405#T2240#c#255312
Copy
Compound
Names
Compound Name
Uridine monophosphate (UMP)
F:\Spectral_Data\..\ESI Neg\Pool18
9/2/2015 5:13:36 AM
Acquity BEH C18
RT: 0.00 - 20.00
1.12
323.0273
1.60
323.0276
NL:
1.25E5
m/z=
323.0269-
323.0301
MS Pool18
Time (min)
323.0273
324.0304
325.0320
323.0286
324.0319
325.0328
326.0362
NL:
1.17E5
Pool18#278 RT:
1.11 AV: 1 T:
FTMS - p ESI Full
ms
[50.00-1200.00]
NL:
8.80E5
pa Chrg -1
m/z

Adenosine monophophate (AMP)

a. Peak not displayed in XCMS Online as p > 0.05.

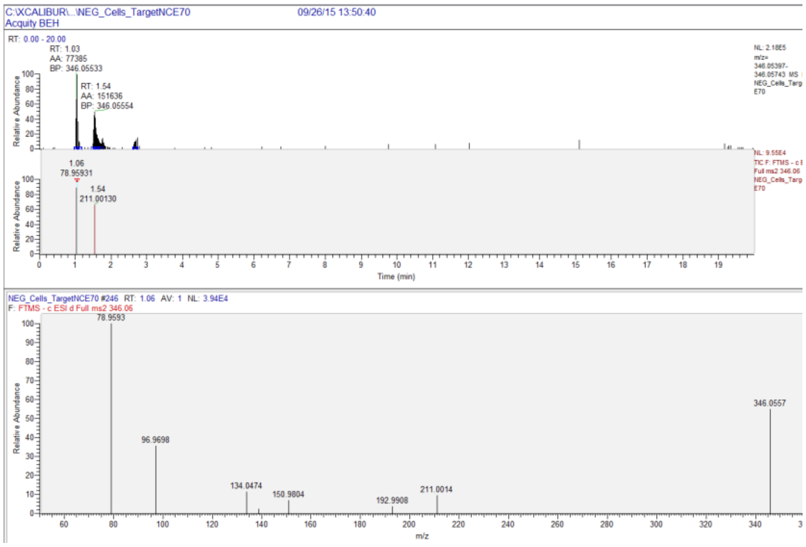

b

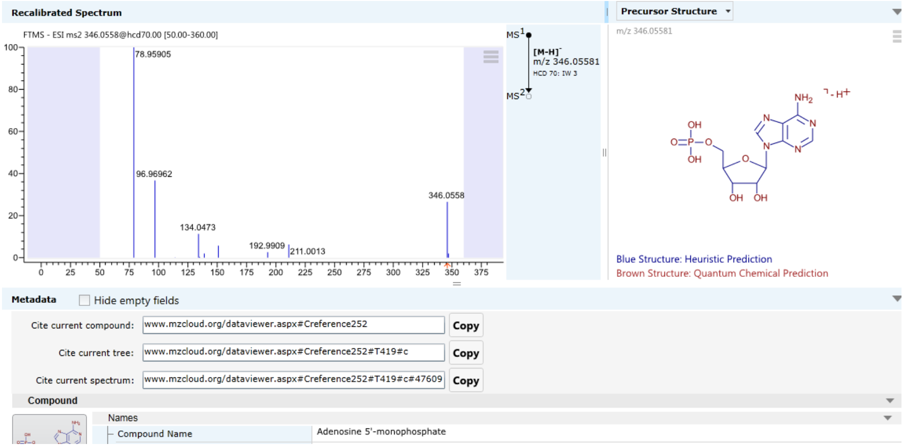

c

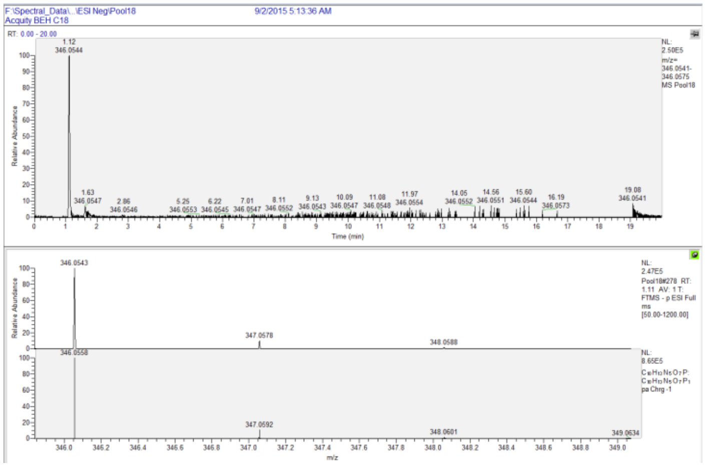

d

Galactonic acid

a. Peak not displayed in XCMS Online as $p > 0.05$.

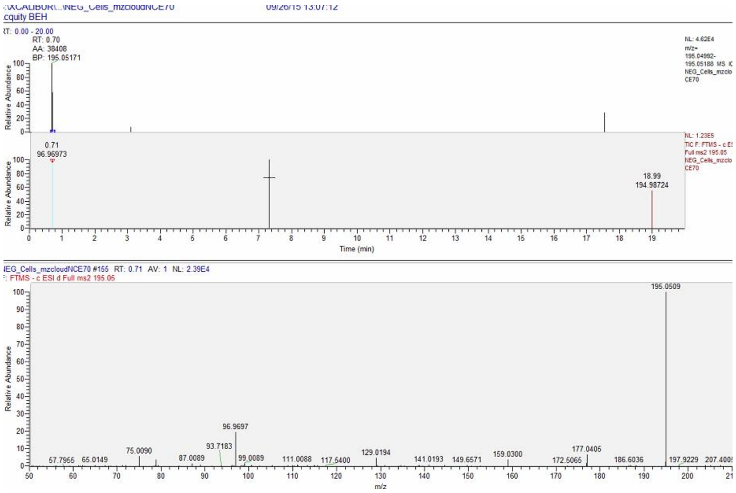

b

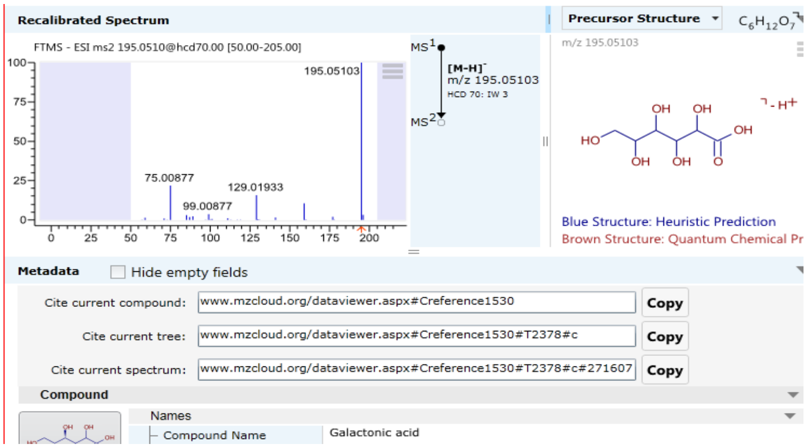

c

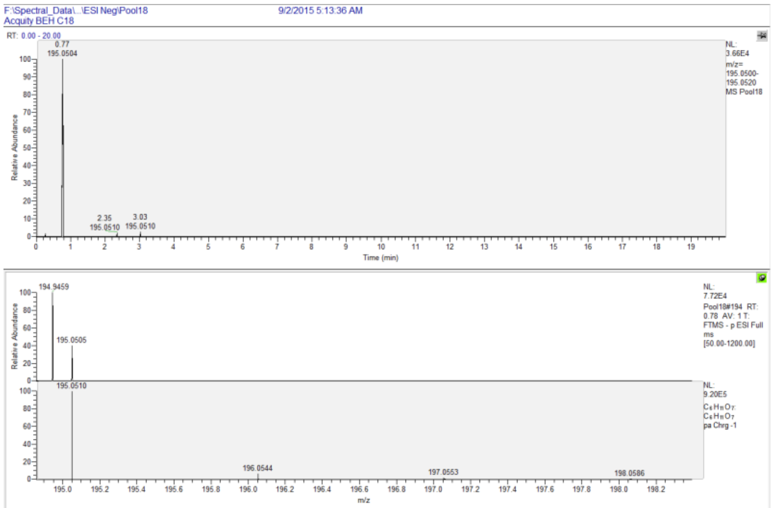

d

Guanosine

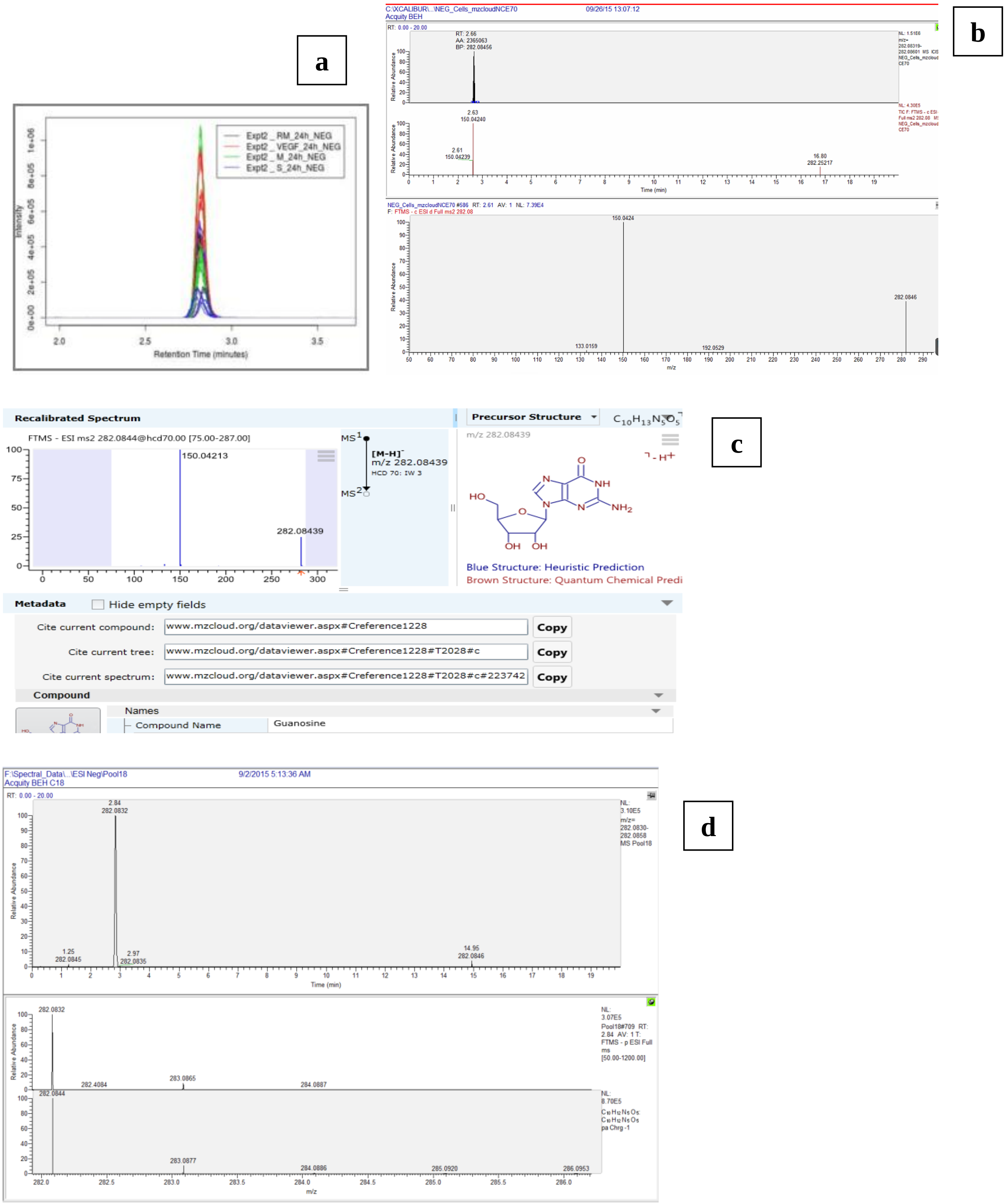
a
Expt2 _ RM_24h_NEG
Expt2 _ VEGF_24h_NEG
Expt2 _ M_24h_NEG
Expt2 _ S_24h_NEG
Intensity
Retention Time (minutes)
b
C:\XCALIBUR\...\NEG_Cells_mzcloudNCE70
09/26/15 13:07:12
Acquity BEH
RT: 0.00 - 20.00
RT: 2.66
AA: 2365063
BP: 282.08456
Relative Abundance
Time (min)
NEG_Cells_mzcloudNCE70 #586 RT: 2.61 AV: 1 NL: 7.39E4
F: FTMS - c ESI d Full ms2 282.08
150.0424
282.0846
133.0159
192.0529
m/z
c
Recalibrated Spectrum
FTMS - ESI ms2 282.0844@hcd70.00 [75.00-287.00]
150.04213
282.08439
MS1
[M-H]⁻
m/z 282.08439
HCD 70: IW 3
MS2
Precursor Structure
C10H13N5O5
m/z 282.08439
Blue Structure: Heuristic Prediction
Brown Structure: Quantum Chemical Predi
Metadata
Hide empty fields
Cite current compound: www.mzcloud.org/dataviewer.aspx#Creference1228
Cite current tree: www.mzcloud.org/dataviewer.aspx#Creference1228#T2028#c
Cite current spectrum: www.mzcloud.org/dataviewer.aspx#Creference1228#T2028#c#223742
Copy
Compound
Names
Compound Name
Guanosine
d
F:\Spectral_Data\...\ESI Neg\Pool18
9/2/2015 5:13:36 AM
Acquity BEH C18
RT: 0.00 - 20.00
2.84
282.0832
NL: 3.10E5
m/z= 282.0830-282.0858 MS Pool18
Relative Abundance
Time (min)
NL: 3.07E5
Pool18#709 RT: 2.84 AV: 1 T: FTMS - p ESI Full ms [50.00-1200.00]
NL: 8.70E5
m/z
