# Supplementary material for: Untargeted metabolomics reveals distinct metabolic reprogramming in endothelial cells co-cultured with CSC and non-CSC prostate cancer cell subpopulations
Source: PLoS One. 2018 Feb 21;13(2):e0192175. doi: 10.1371/journal.pone.0192175 (PMC5821452; doi:10.1371/journal.pone.0192175)
Supplement: S2 Fig — (DOCX) [file pone.0192175.s003.docx]

**S2 Fig:** Pathways generated with identified metabolites in HUVEC in the presence of VEGF, PC-3/S and PC-3/M cells, by metabolite set enrichment analysis using MetaboAnalyst 3.0.
