# Supplementary material for: Untargeted metabolomics reveals distinct metabolic reprogramming in endothelial cells co-cultured with CSC and non-CSC prostate cancer cell subpopulations
Source: PLoS One. 2018 Feb 21;13(2):e0192175. doi: 10.1371/journal.pone.0192175 (PMC5821452; doi:10.1371/journal.pone.0192175)
Supplement: S3 Fig — (DOCX) [file pone.0192175.s004.docx]

**S3 Fig:** The fold change ratio of the VEGF-A gene expression data, showing VEGF-A expressed by PC-3S to PC-3M cells, where PC-3S cells show higher expression relative to PC-3M cells.

**
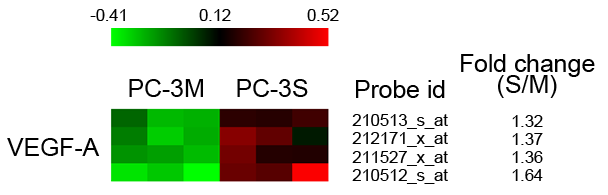
**
